# Supplementary material for: A Plant-Based Diet Alleviates Molecular Pulmonary Abnormalities in Hypertension
Source: Adv Respir Med. 2025 Nov 4;93(6):49. doi: 10.3390/arm93060049 (PMC12641640; doi:10.3390/arm93060049)

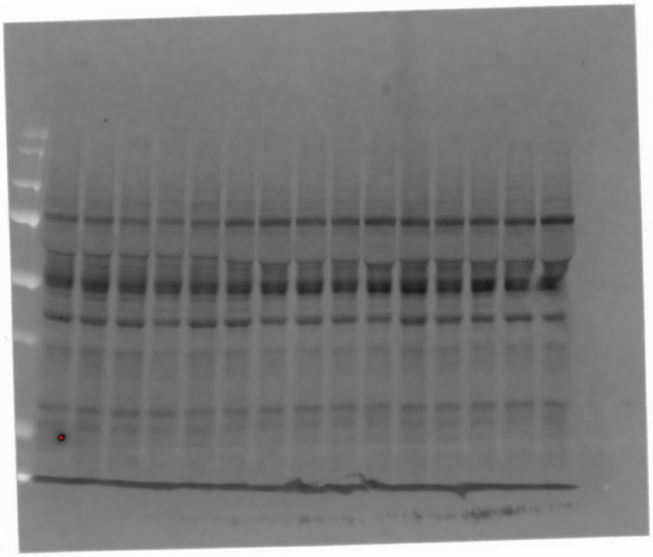

Total lung membrane protein for 40-week-old rats (treatment phase) which was probed for: p38, NRF2, and eNOS.

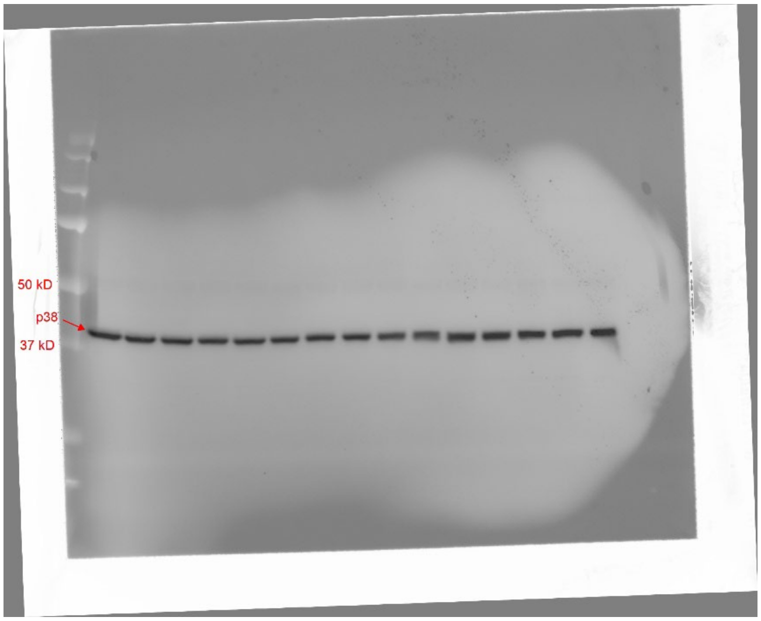
50 kD
p38
37 kD

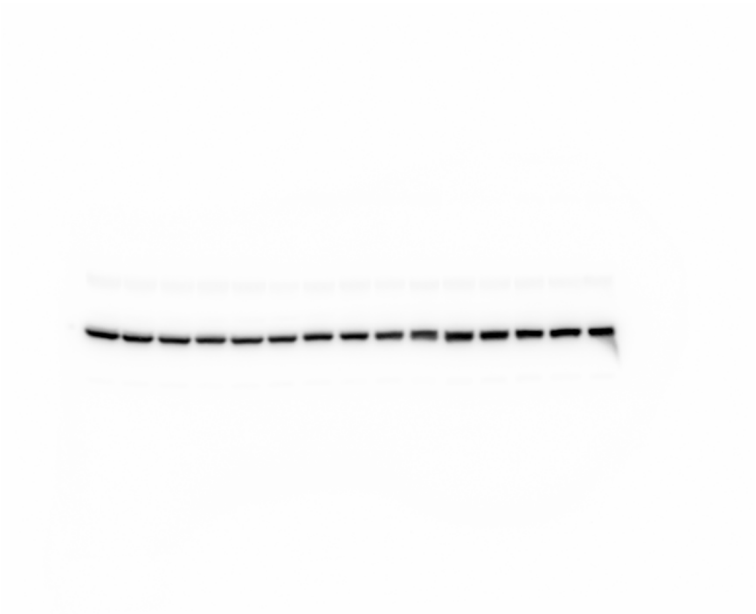

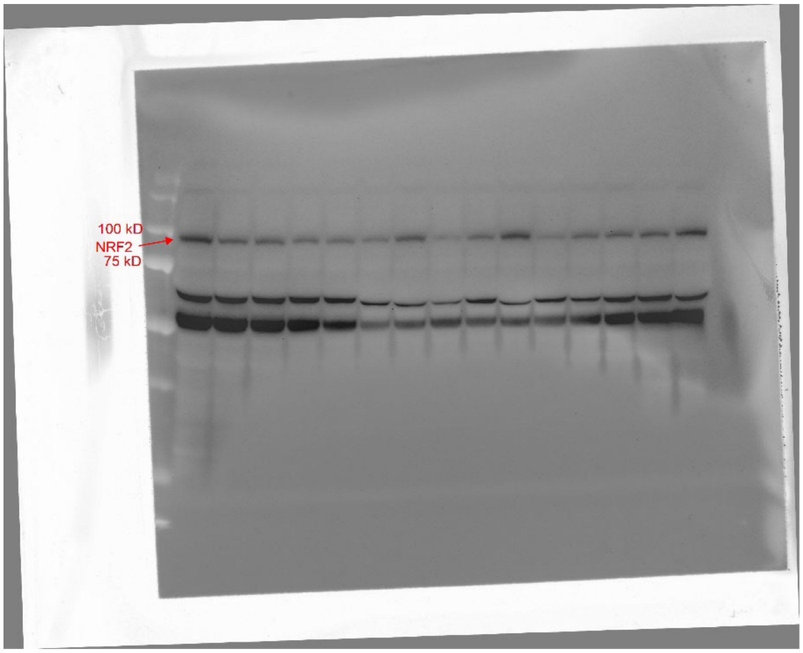

100 kD
NRF2
75 kD

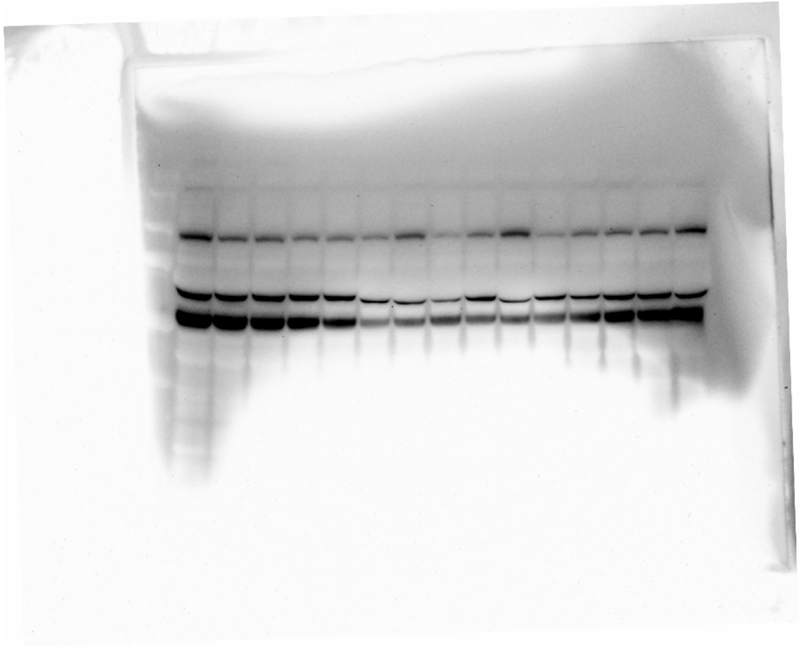

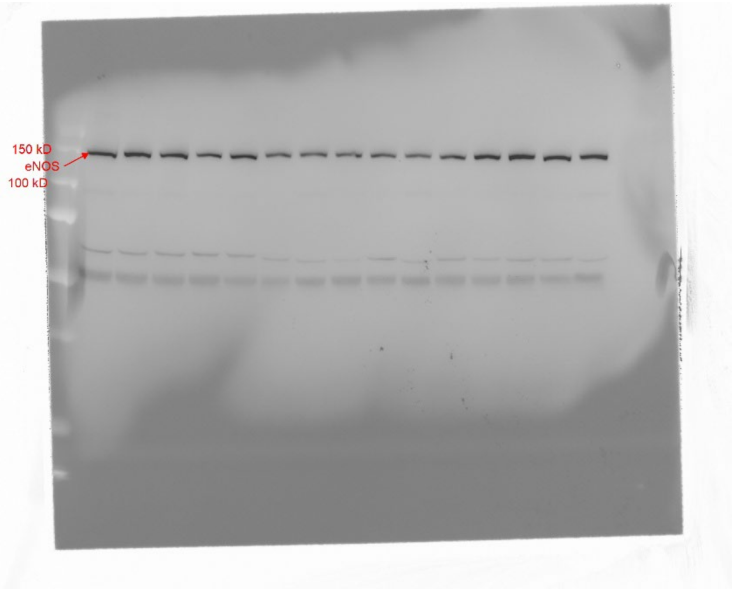
150 kD
eNOS
100 kD

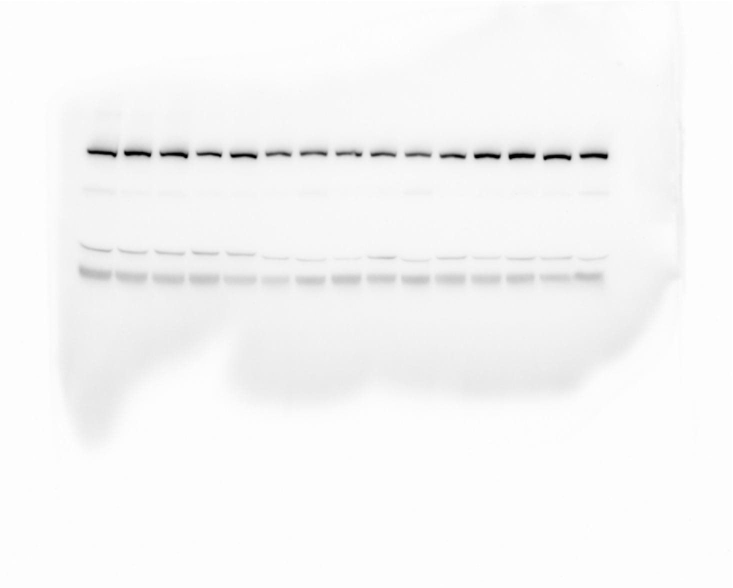

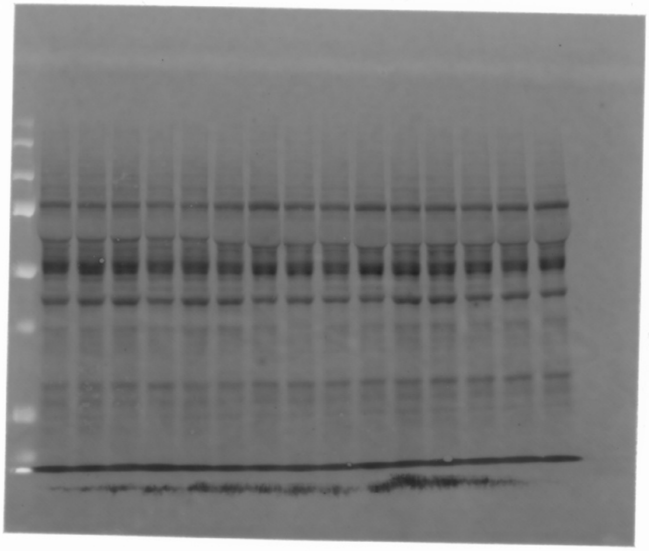

Total lung membrane protein for 40-week-old rats (treatment phase) which was probed for: SOD2, p-SAPK, p-p38, and E-cadherin.

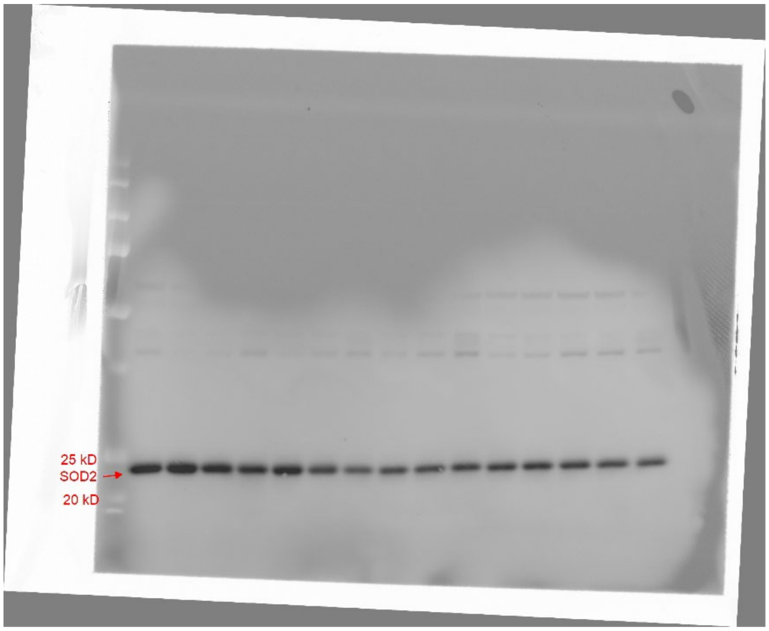

25 kD
SOD2
20 kD

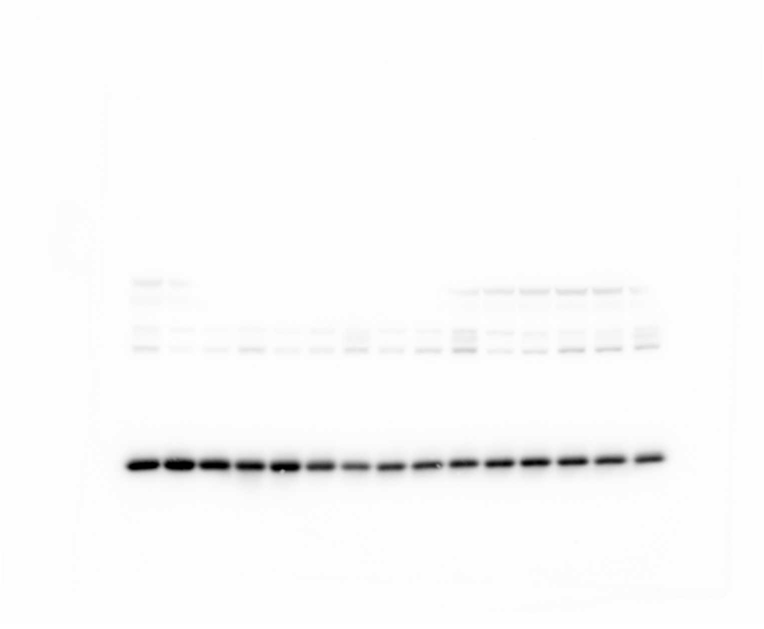

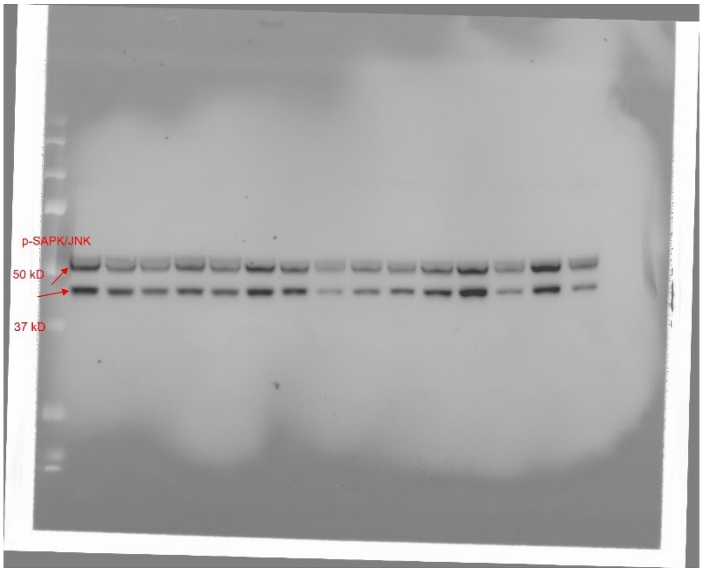
p-SAPK/JNK
50 kD
37 kD

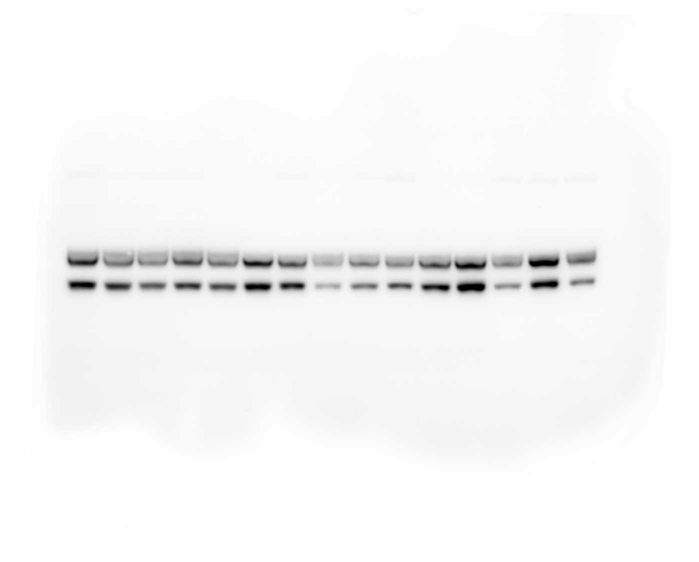

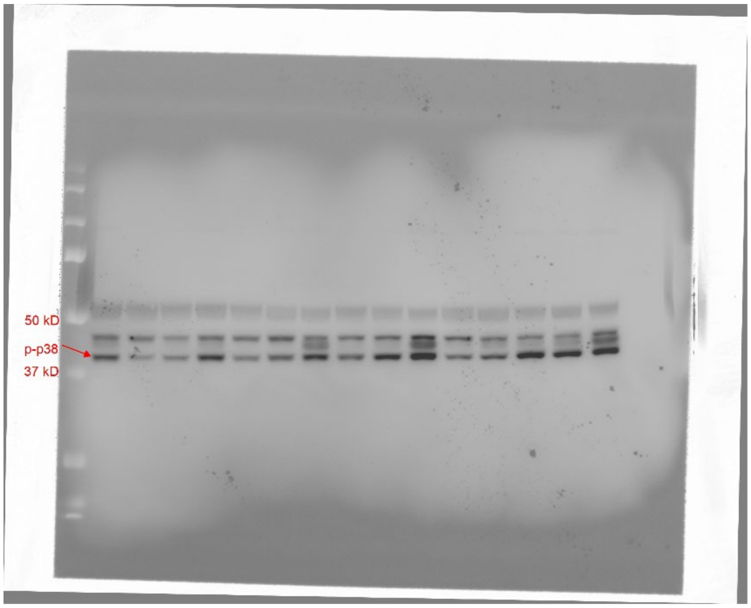

50 kD
p-p38
37 kD

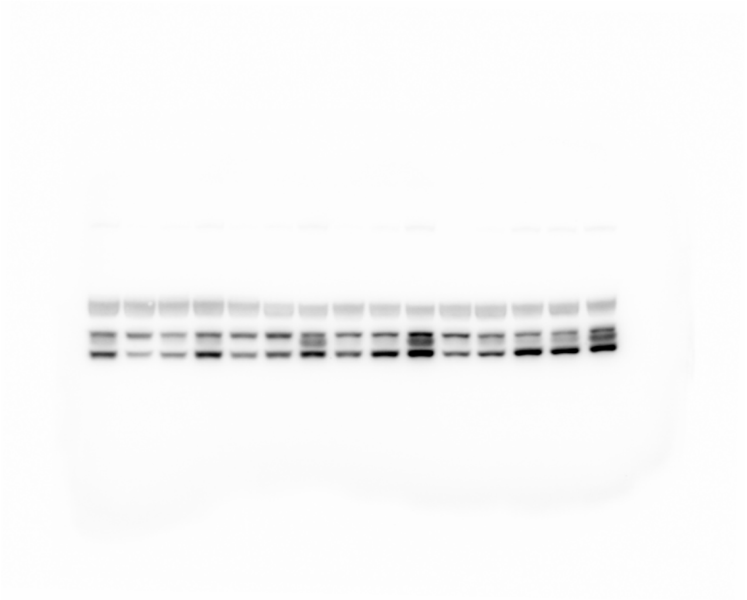

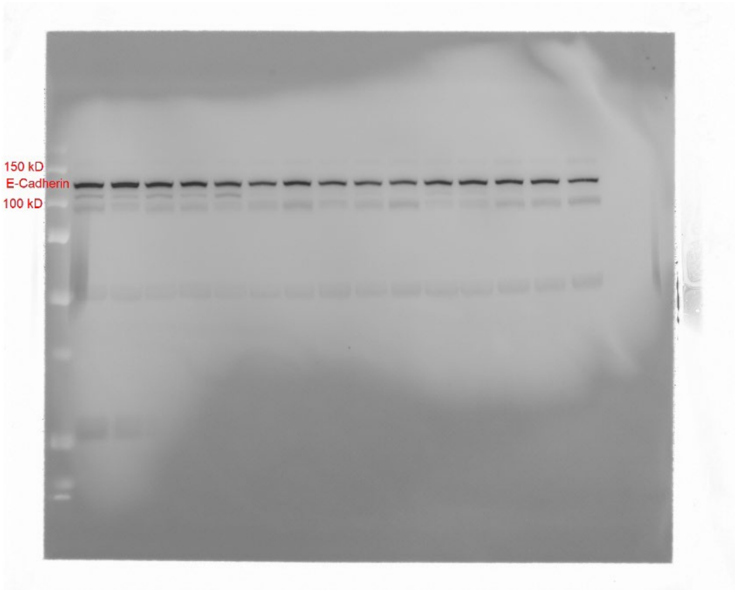
150 kD
E-Cadherin
100 kD

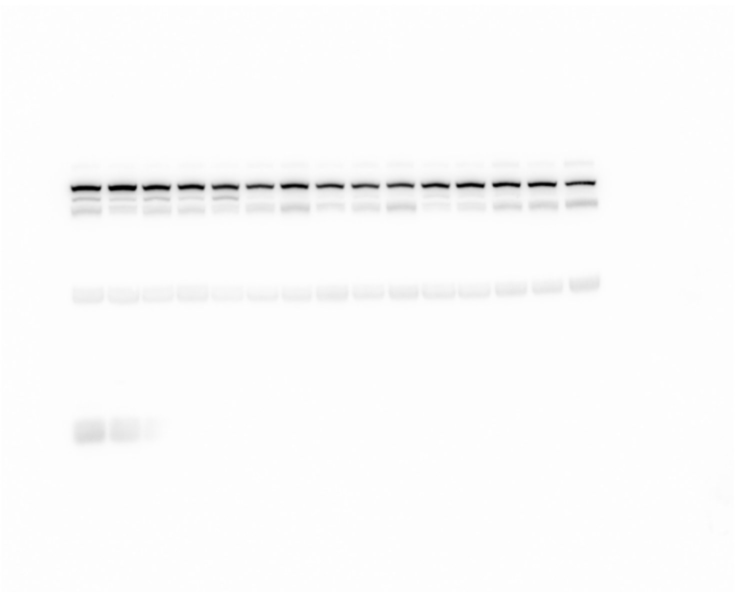

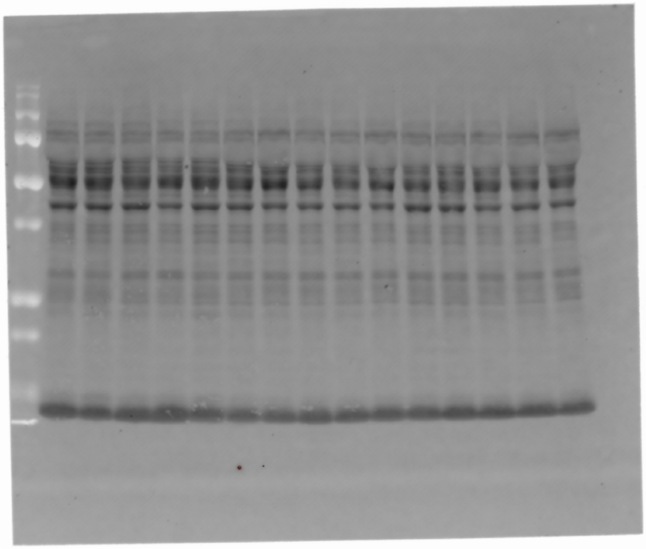

Total lung membrane protein for 40-week-old rats (treatment phase) which was probed for: p-NF-κB, p47phox, p22phox, and c-Jun.

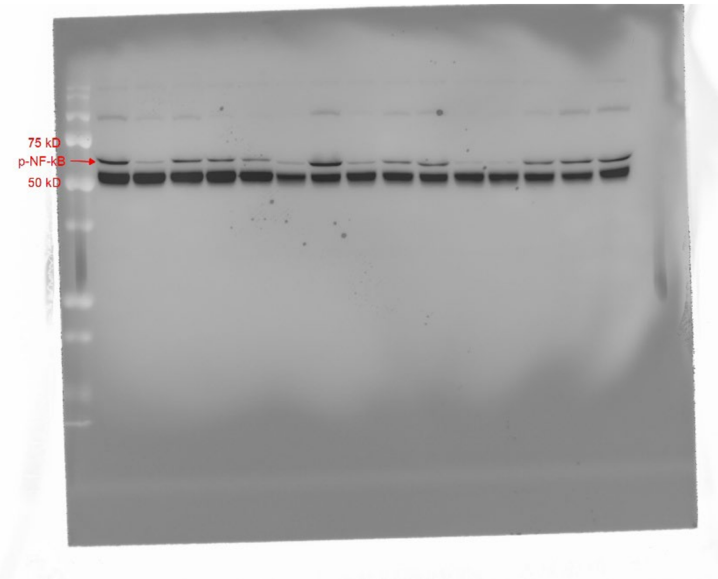
75 kD
p-NF-kB
50 kD

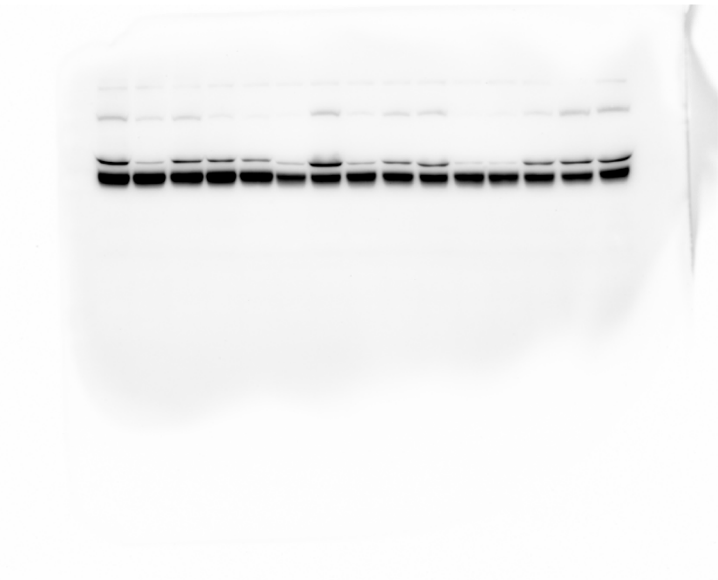

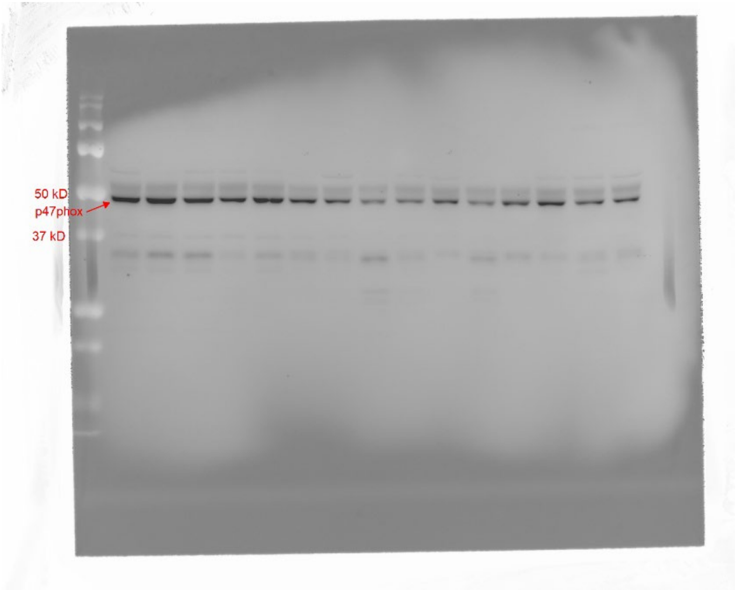

50 kD
p47phox
37 kD

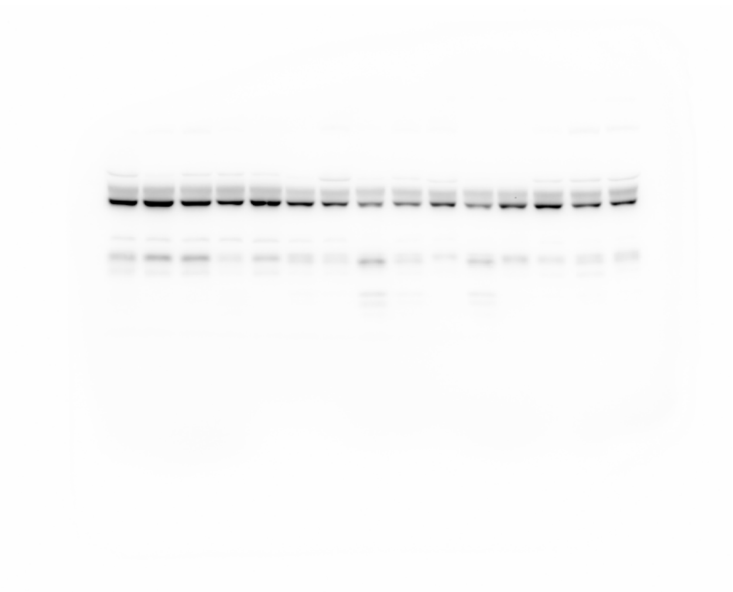

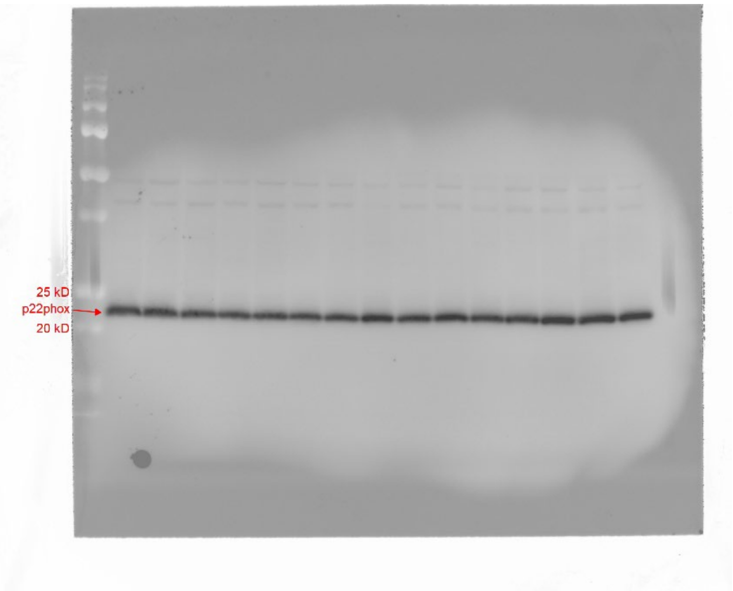

25 kD
p22phox
20 kD

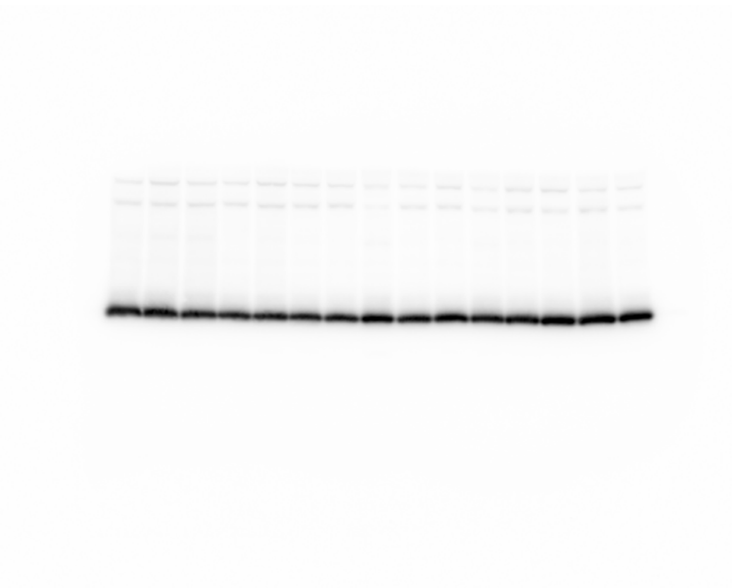

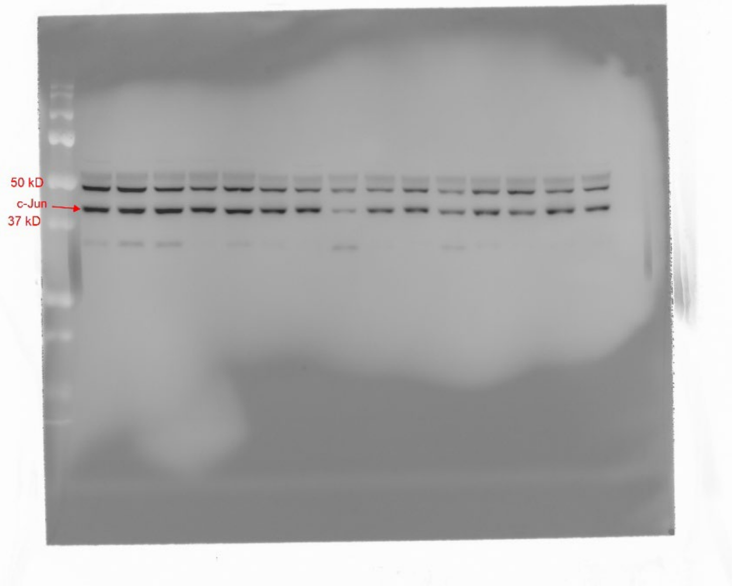
50 kD
c-Jun
37 kD

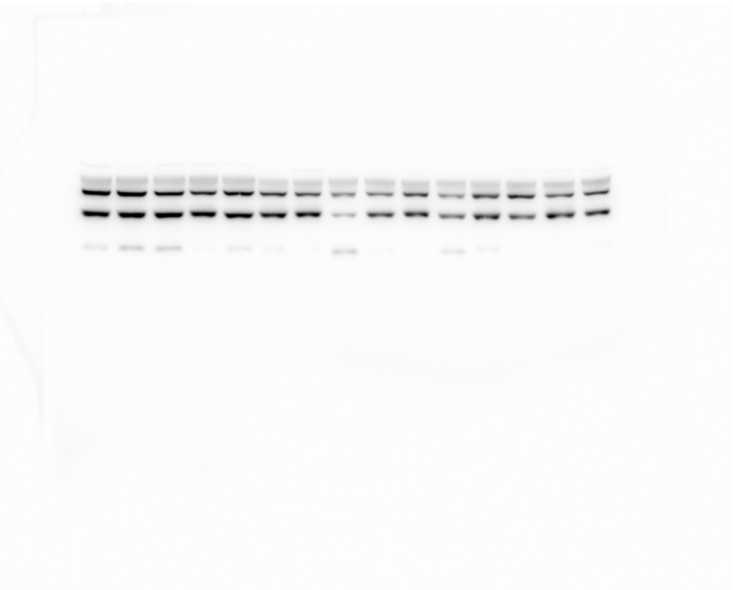

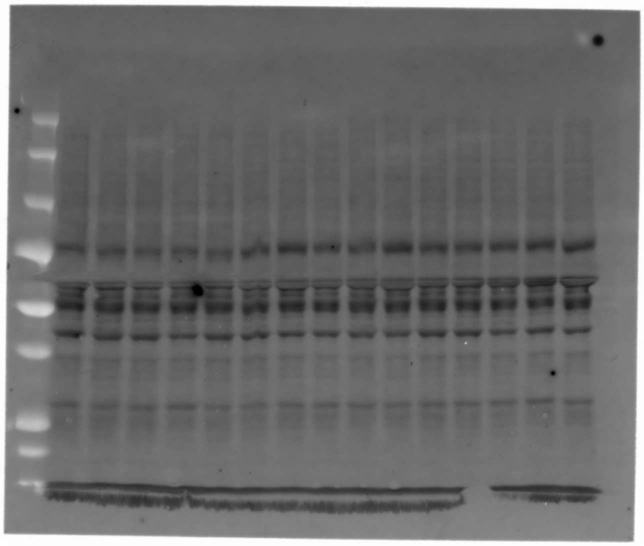

Total lung membrane protein for 40-week-old rats (treatment phase)which was probed for: p-c-Jun and F4/80

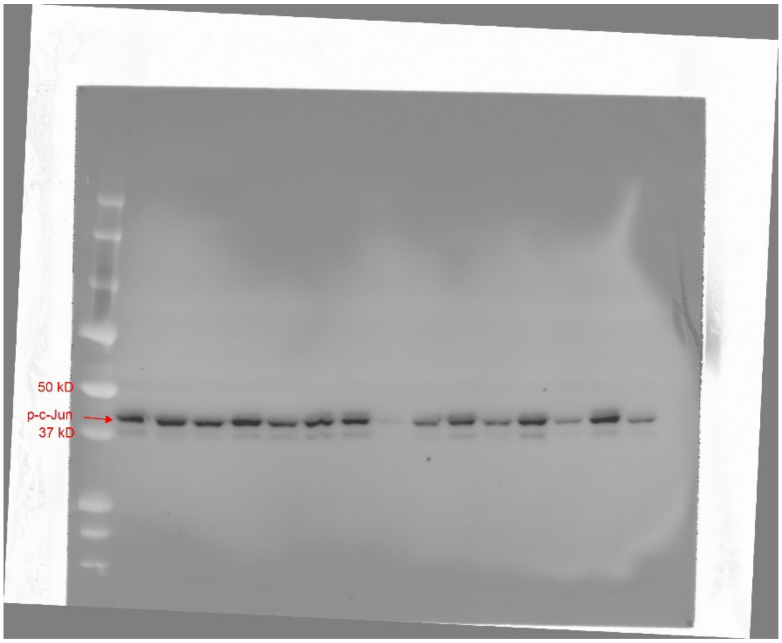
50 kD
p-c-Jun
37 kD

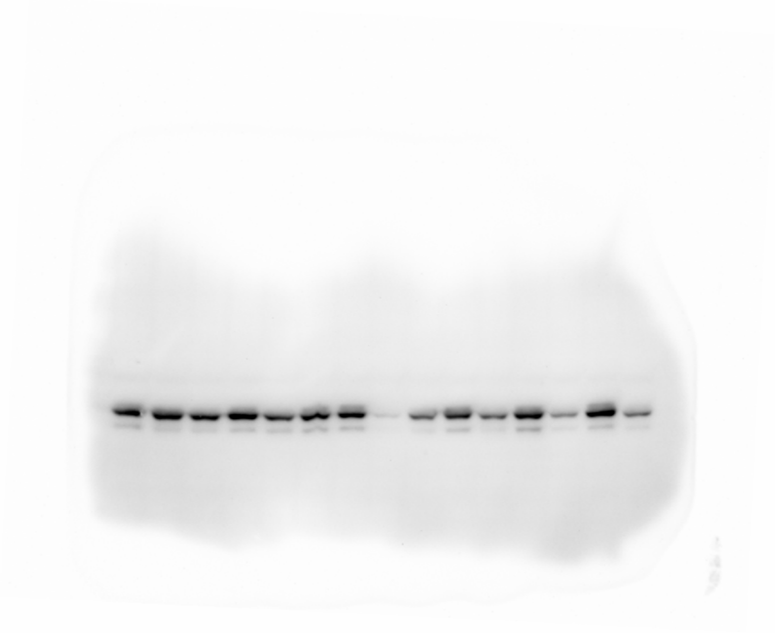

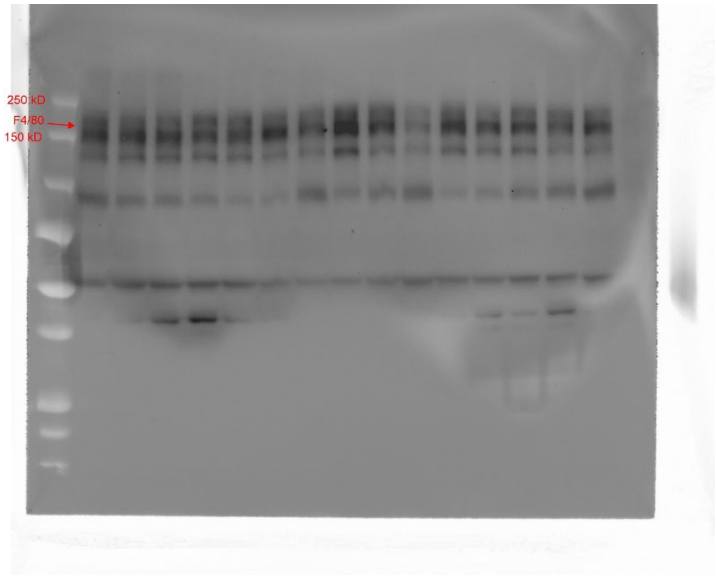
250 kD
F4/80
150 kD

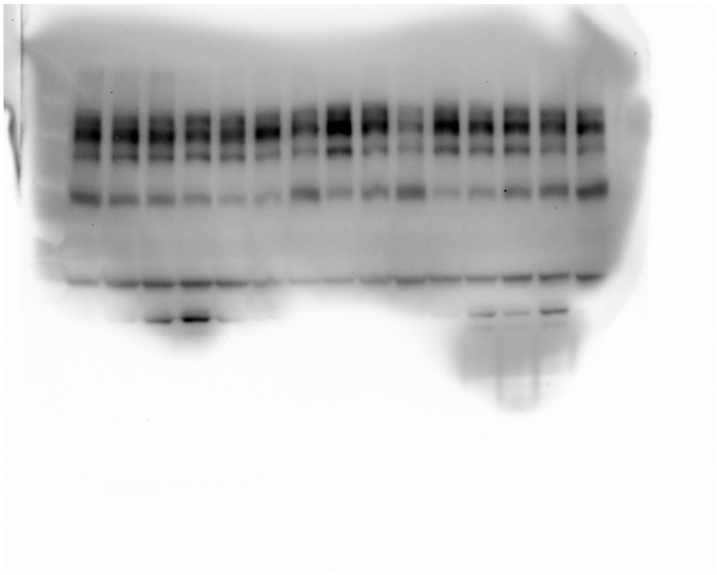

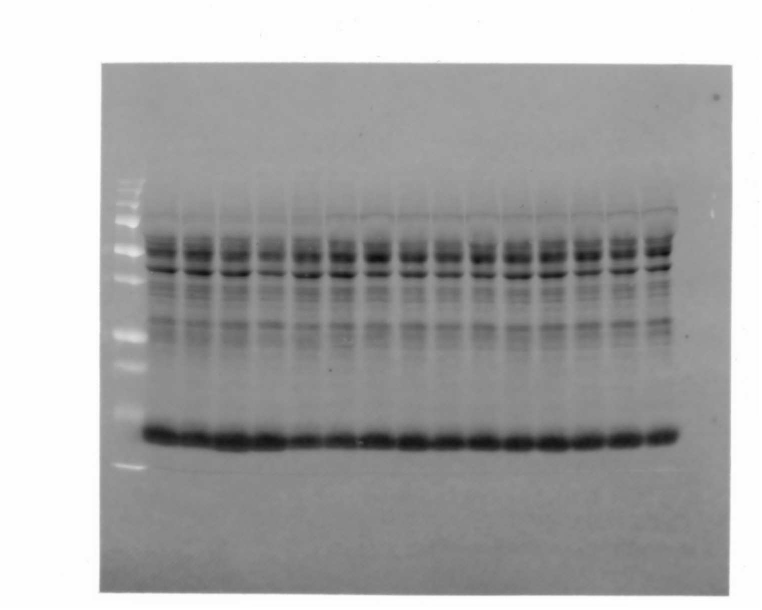

Total lung membrane protein for 40-week-old rats (treatment phase) which was probed for: cleaved and pro TGF-B1

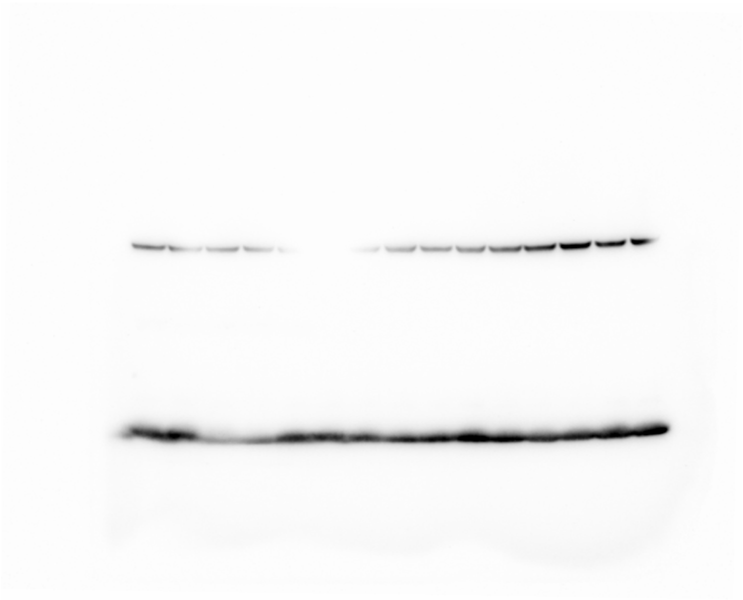

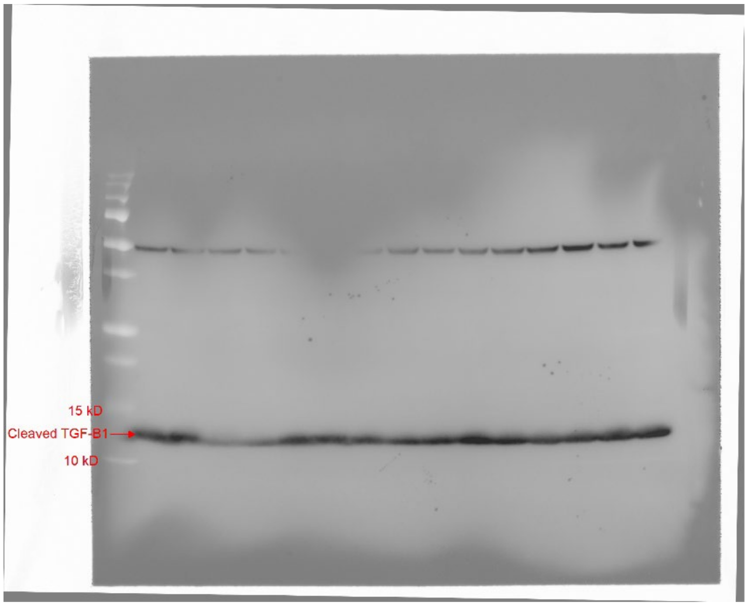
15 kD
Cleaved TGF-B1
10 kD

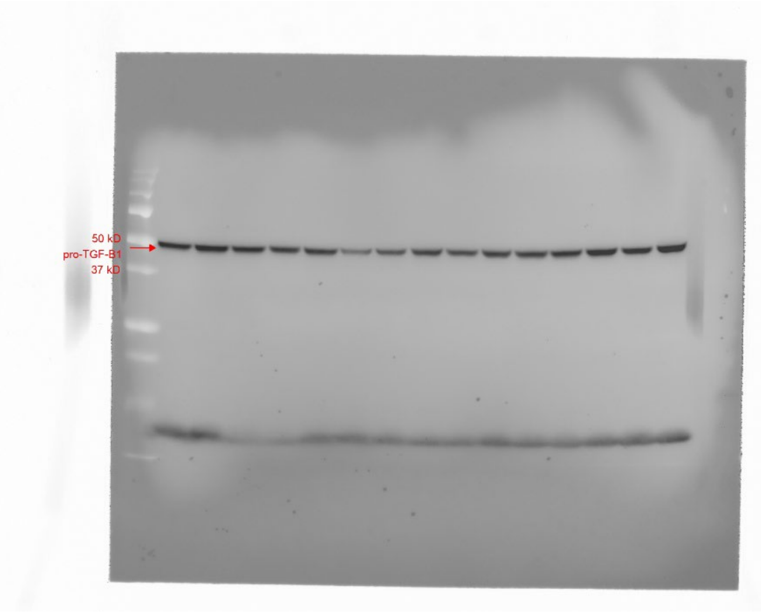

50 kD
pro-TGF-B1
37 kD

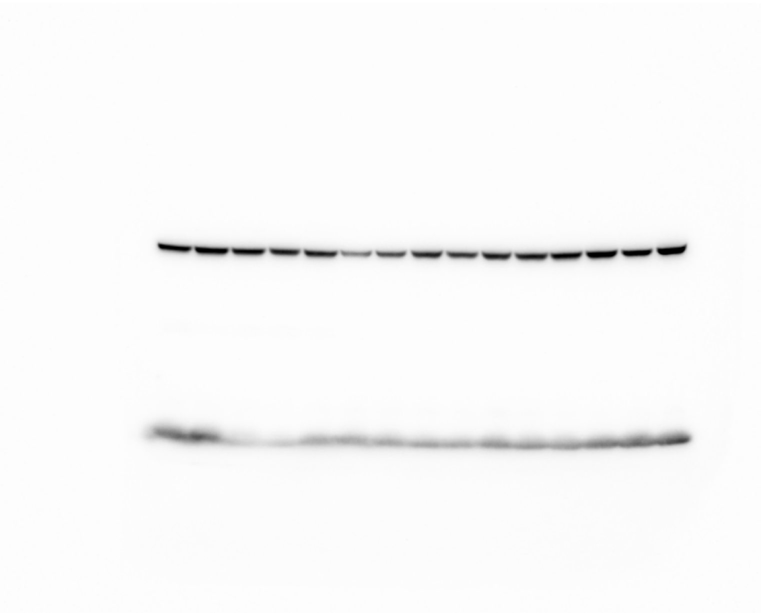

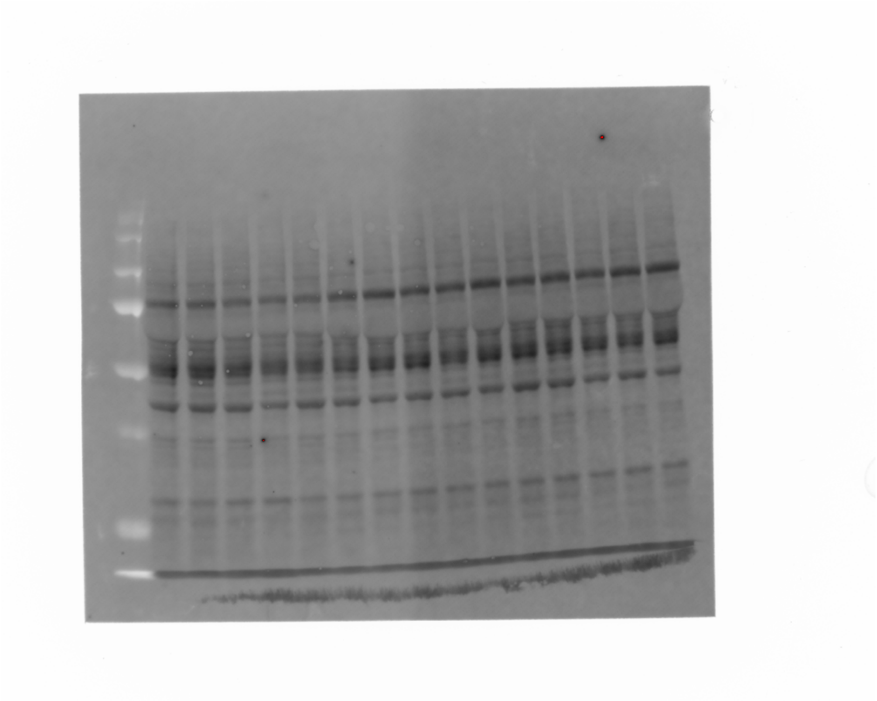

Total lung membrane protein for 40-week-old rats (treatment phase) which was probed for: CAT, ERK1/2, and SAPK/JNK

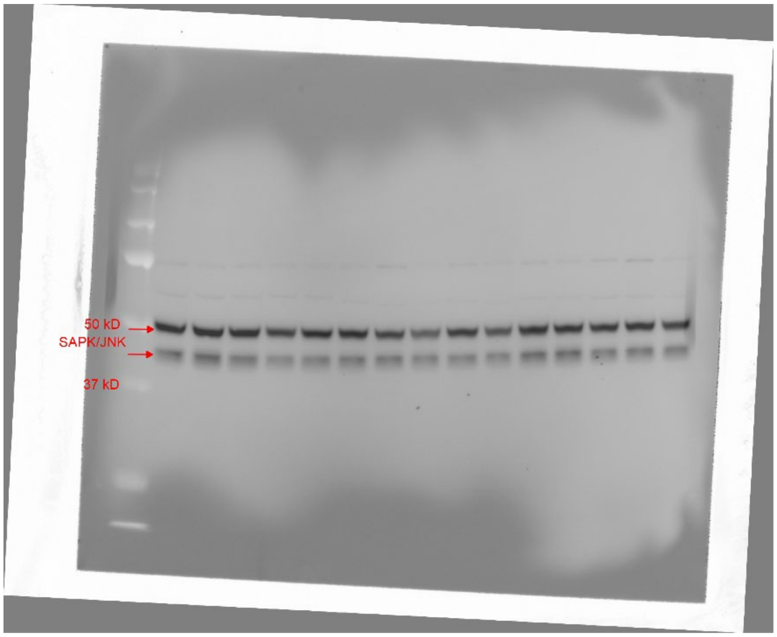
50 kD
SAPK/JNK
37 kD

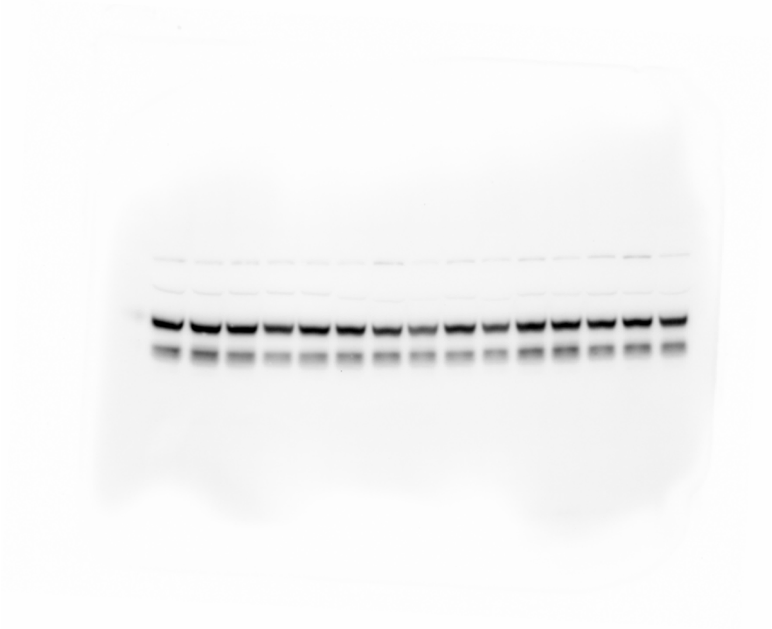

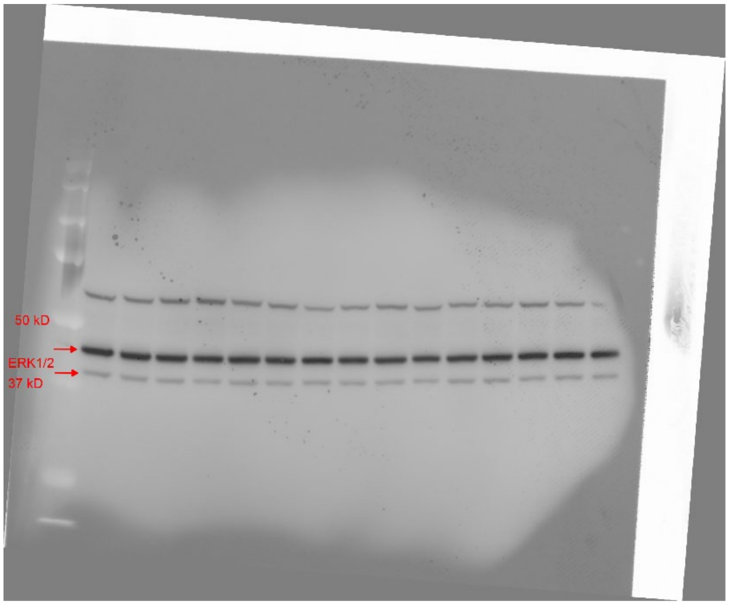
50 kD
ERK1/2
37 kD

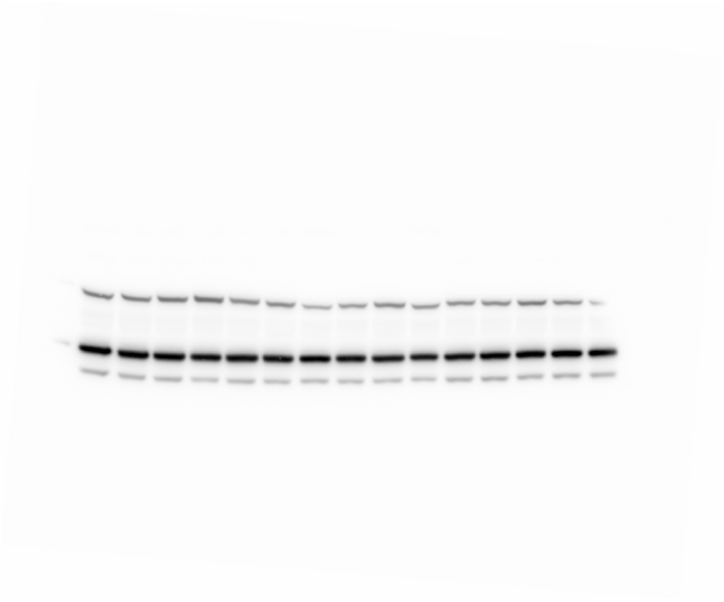

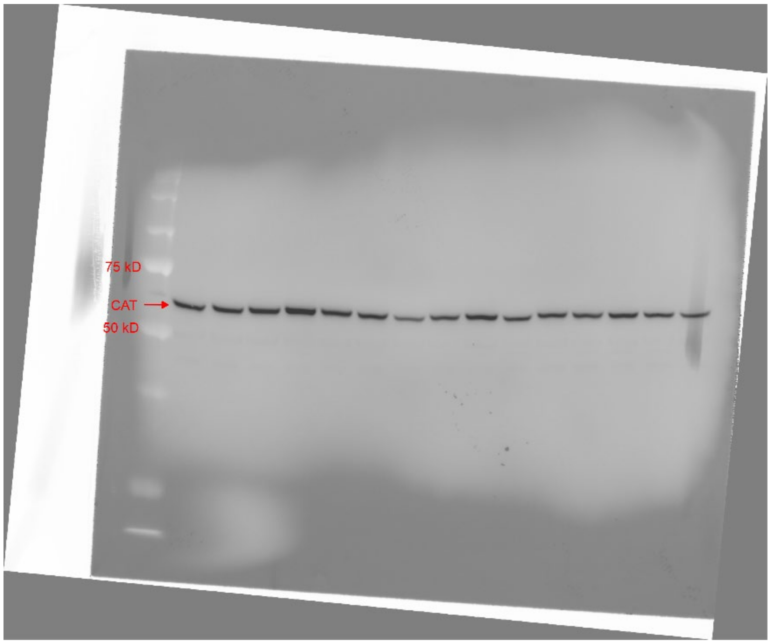
75 kD
CAT
50 kD

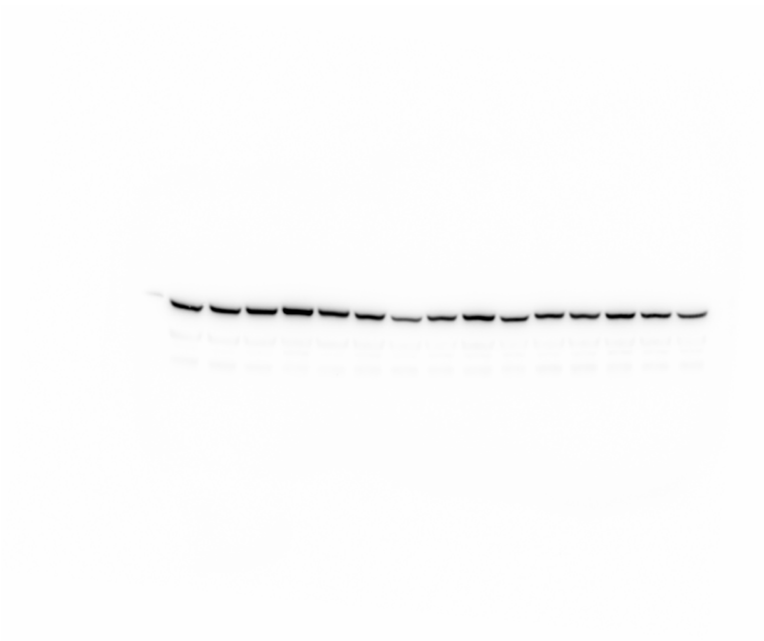

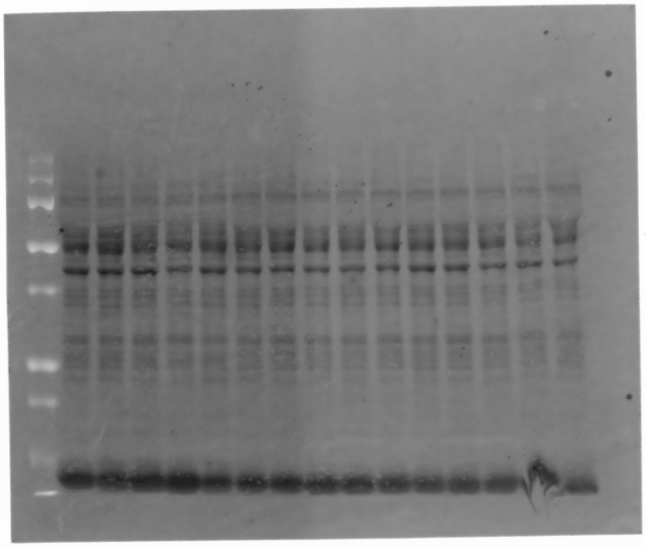

Total lung membrane protein for 40-week-old rats (treatment phase) which was probed for: p-ERK1/2 and NF-κB

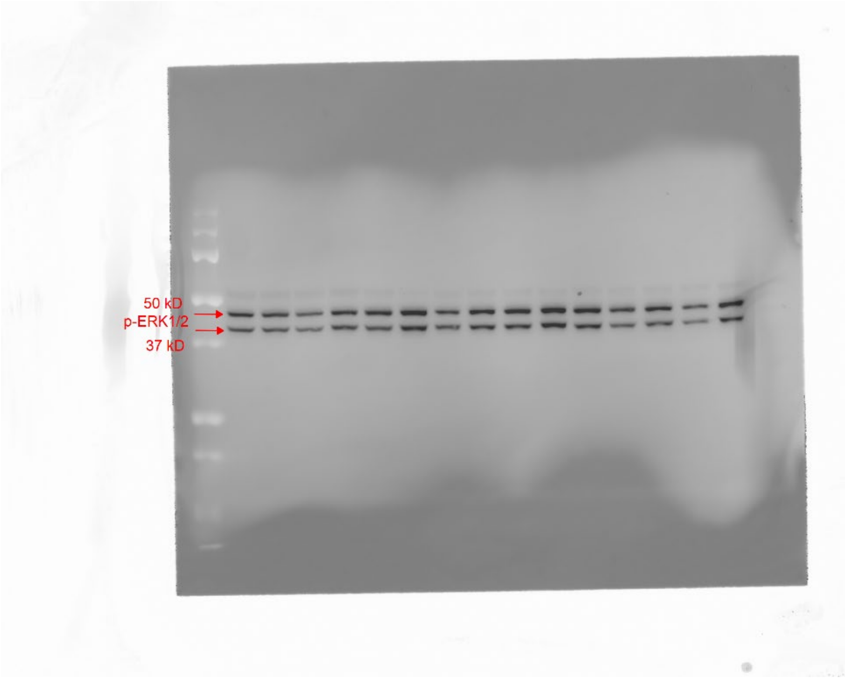
50 kD
p-ERK1/2
37 kD

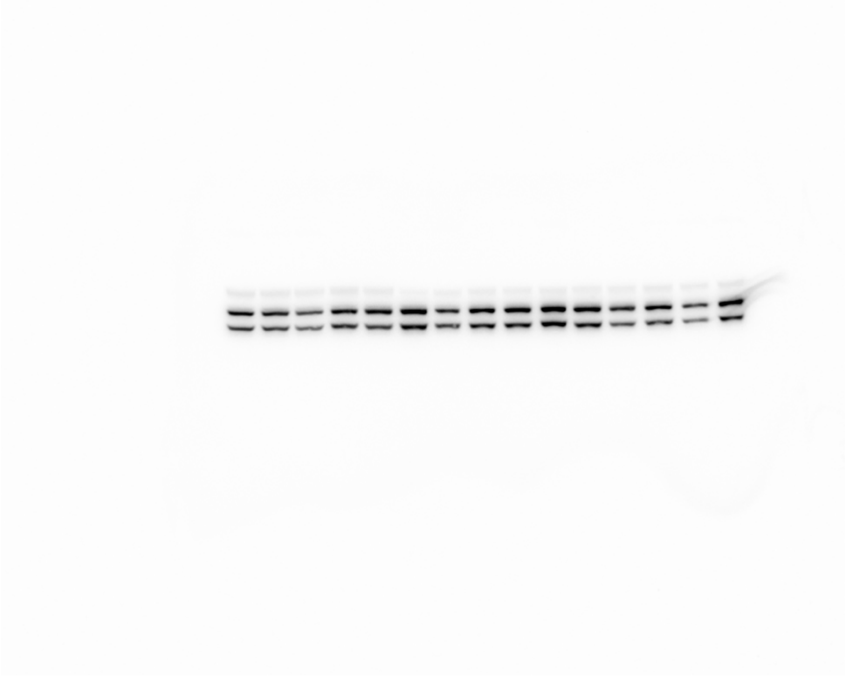

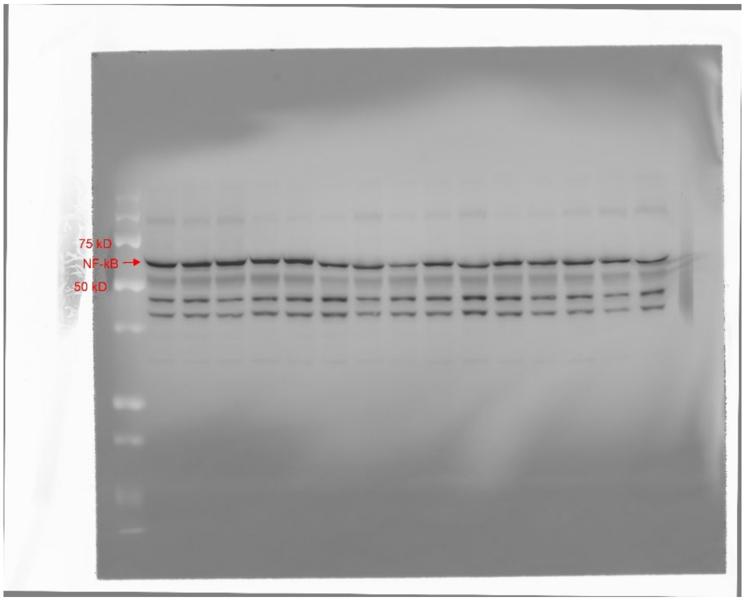
75 kD
NF-kB
50 kD

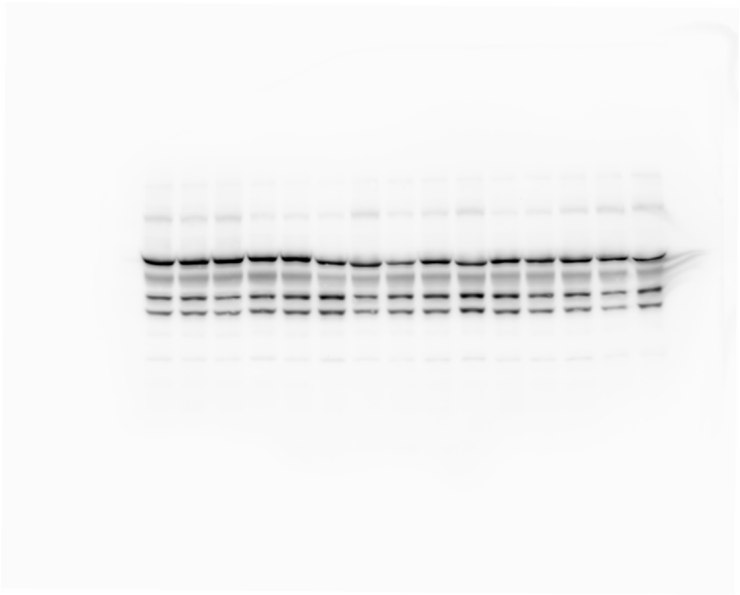

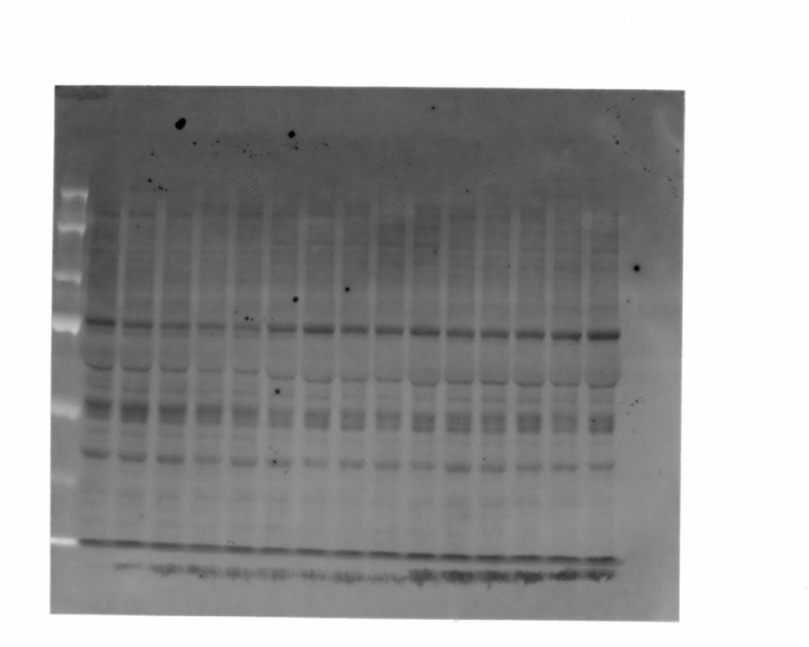

Total lung membrane protein for 40-week-old rats (treatment phase) which was probed for: Vimentin, α-SMA, and MMP-9.

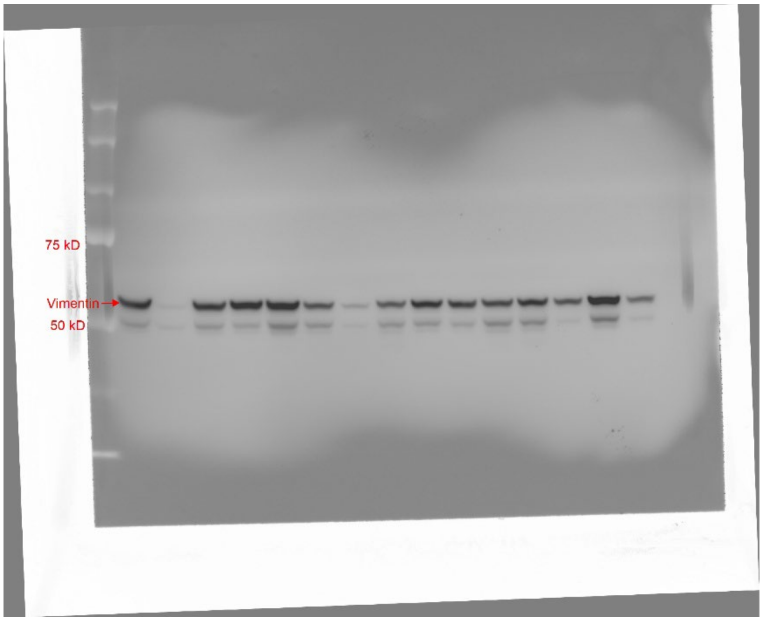
75 kD
Vimentin
50 kD

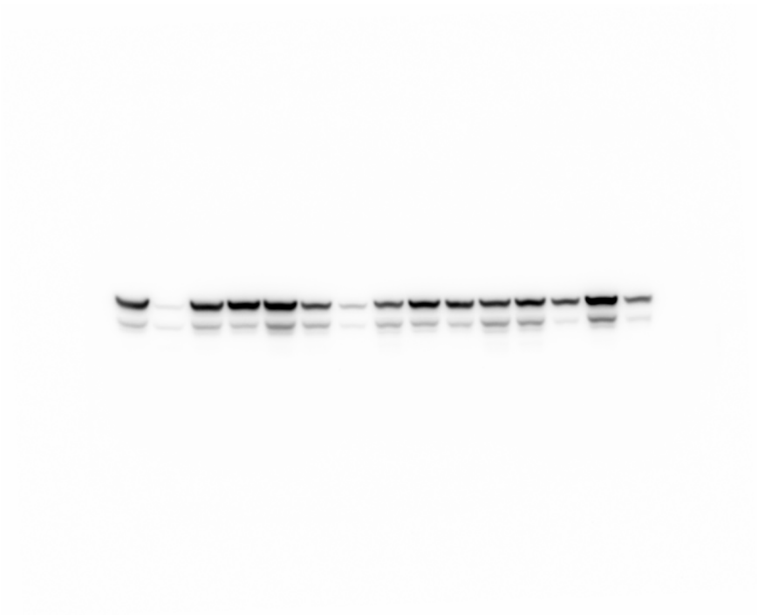

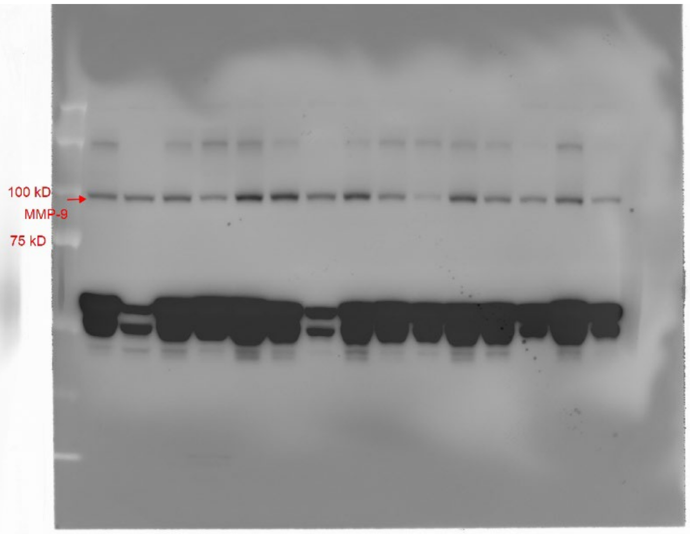

100 kD
MMP-9
75 kD

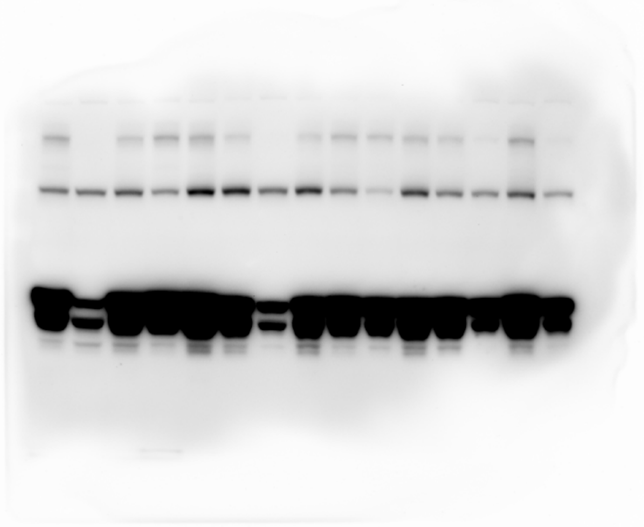

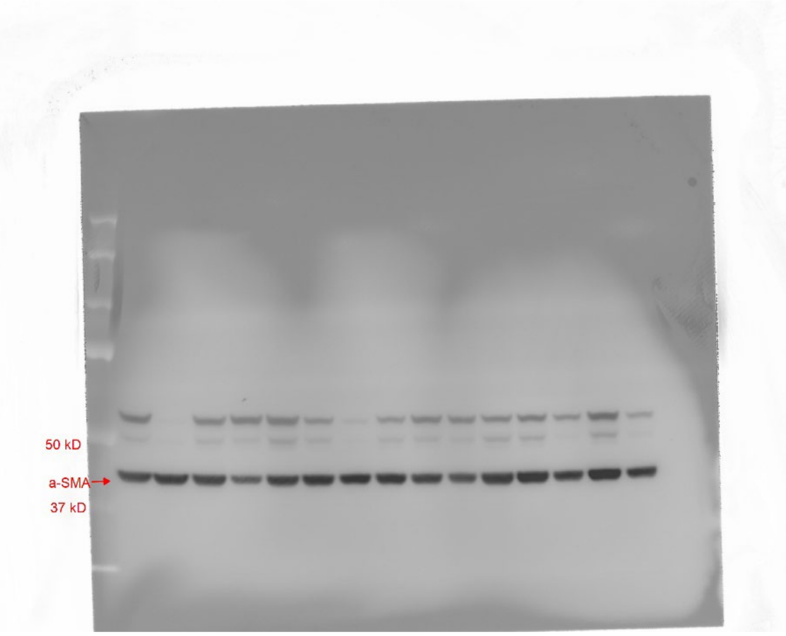
50 kD
a-SMA
37 kD

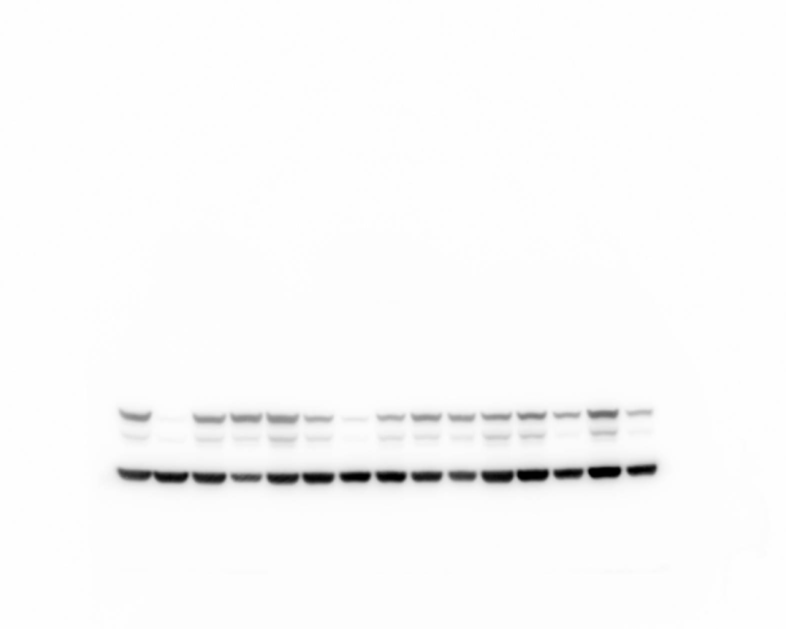

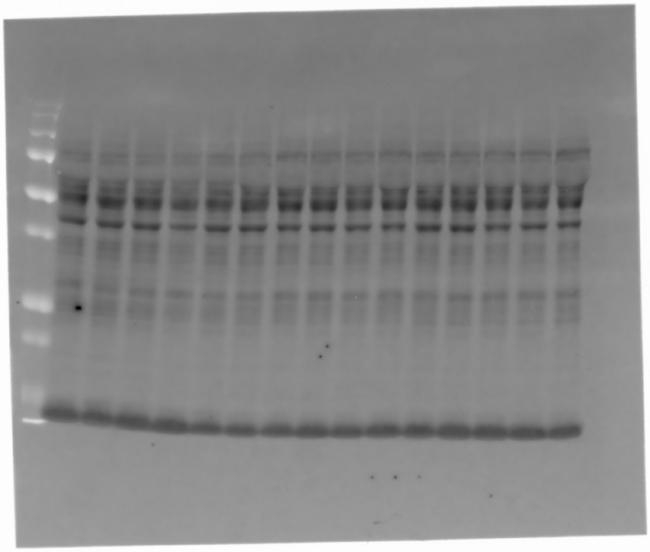

Total lung membrane protein for 40-week-old rats (treatment phase) which was probed for: GPx1, 3-NT, SOD1, and XO.

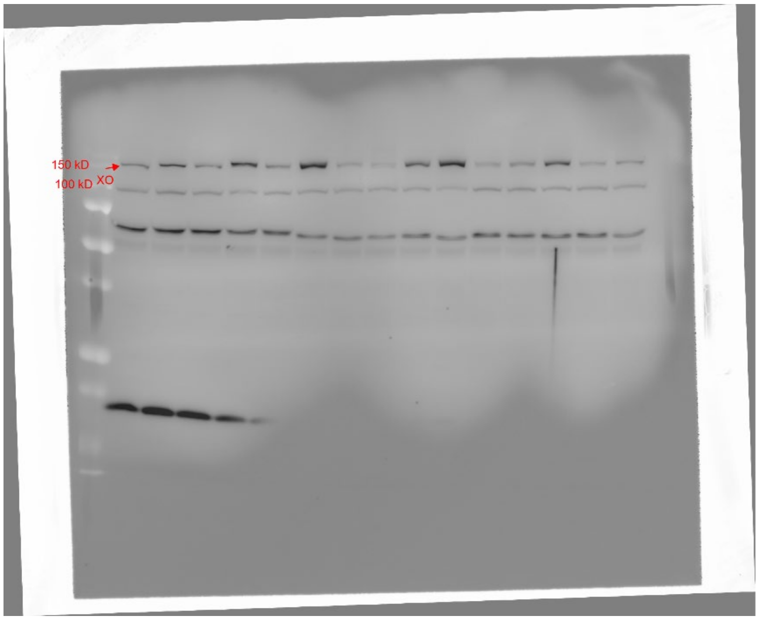
150 kD
100 kD
XO

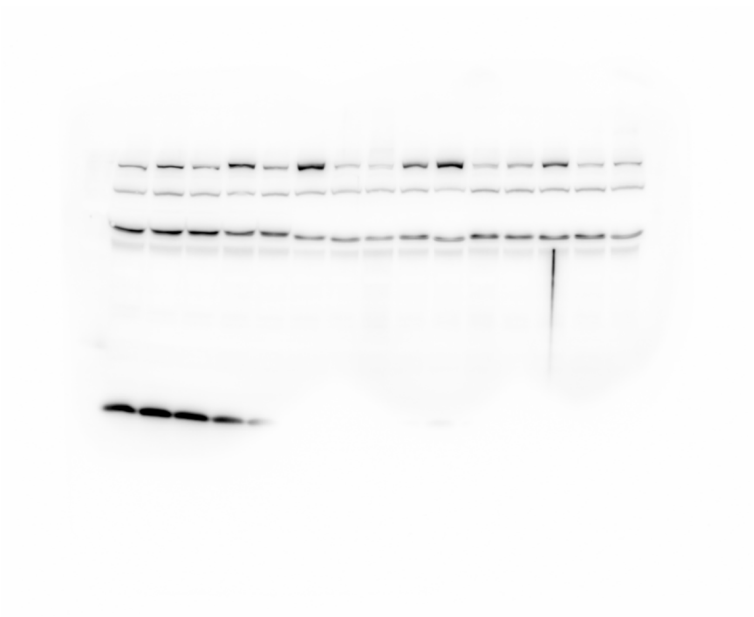

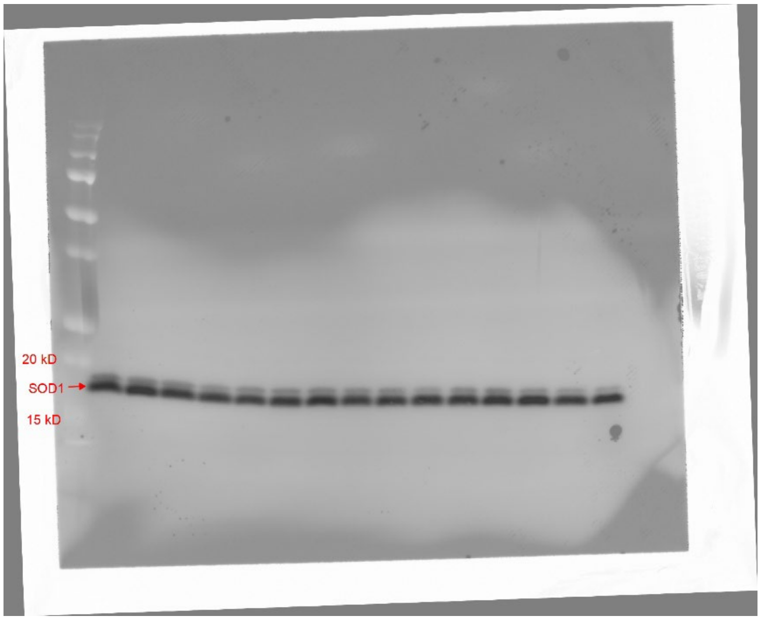
20 kD
SOD1
15 kD

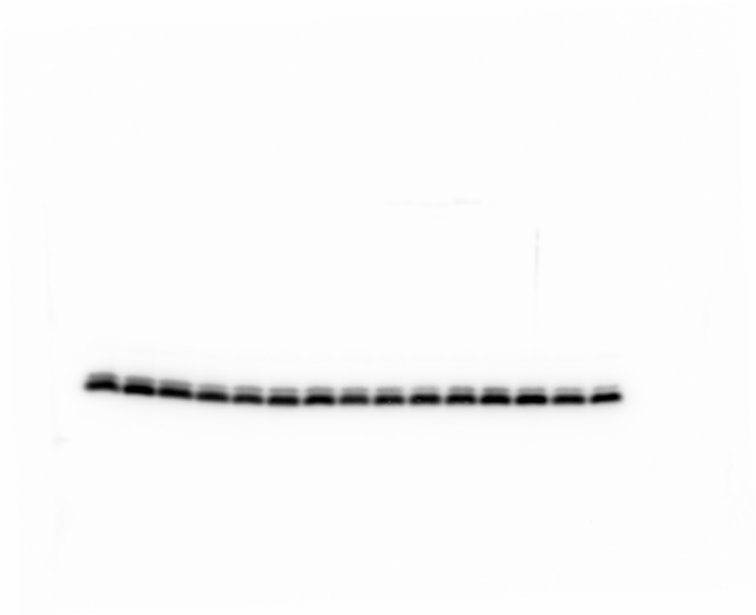

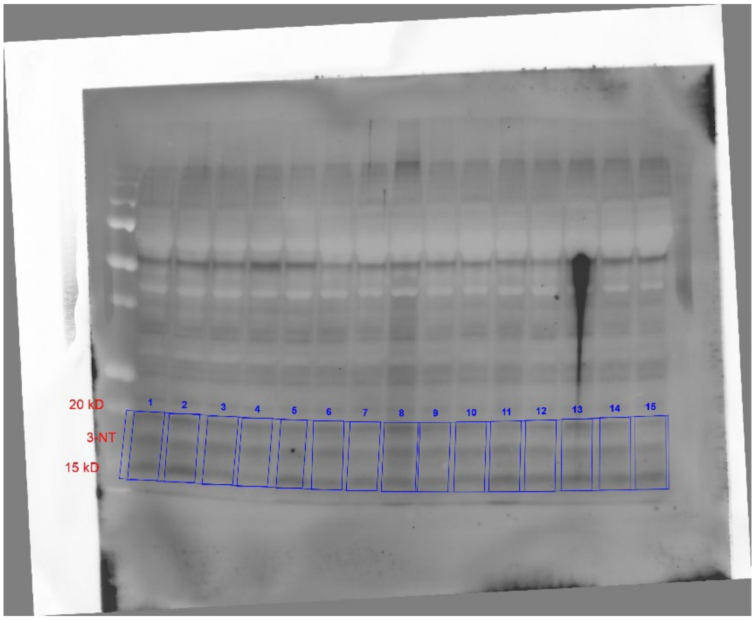

20 kD
3-NT
15 kD
1
2
3
4
5
6
7
8
9
10
11
12
13
14
15

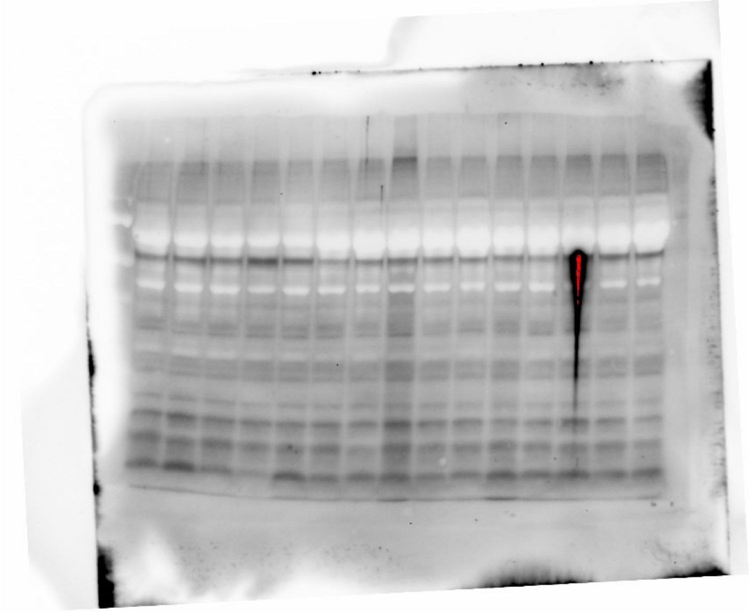

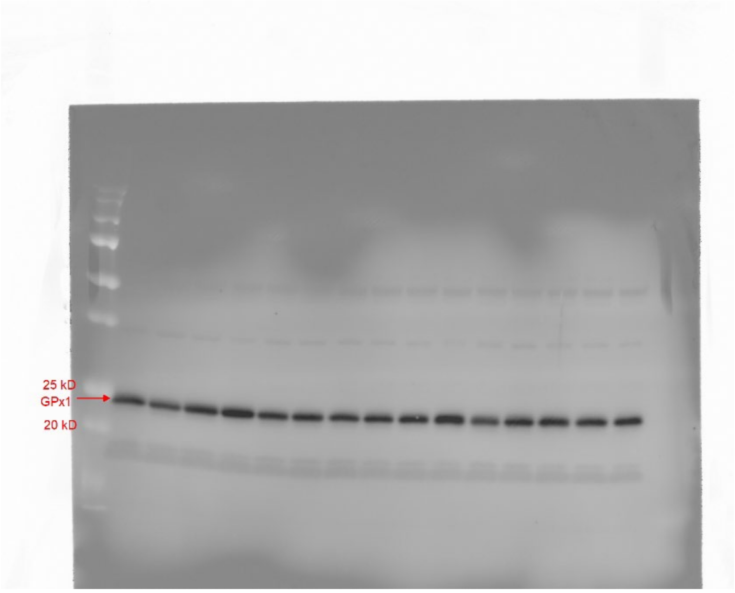
25 kD
GPx1
20 kD

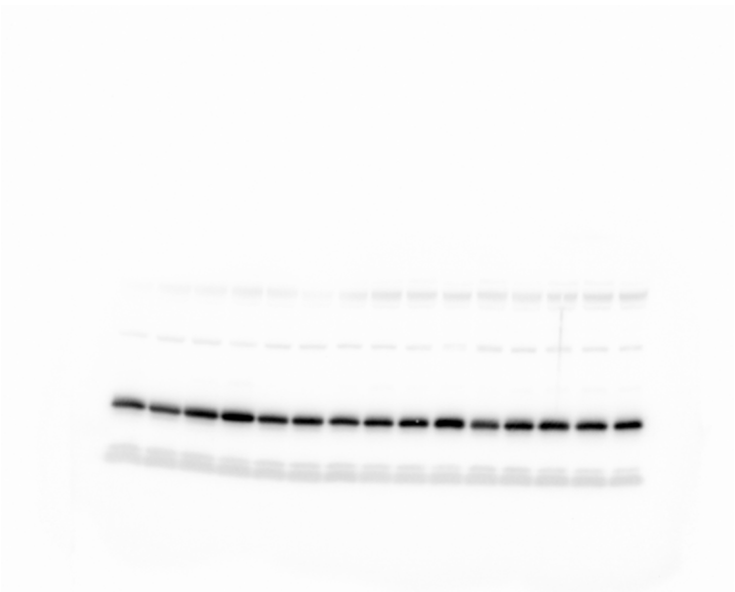

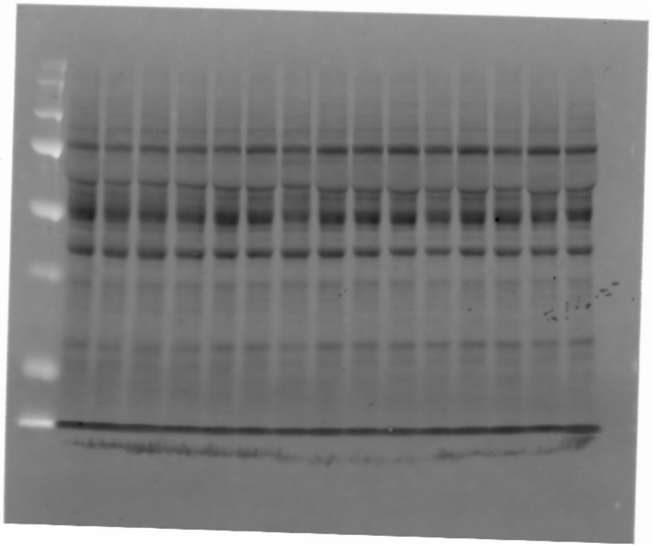

Total lung membrane protein for 28-week-old rats (prevention phase) which was probed for: p47phox and c-Jun

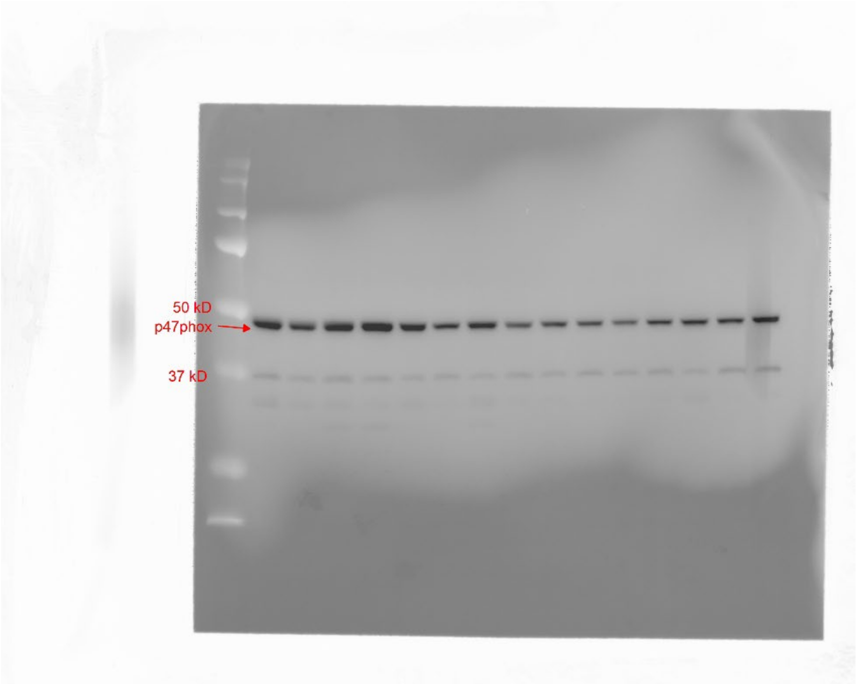

50 kD
p47phox
37 kD

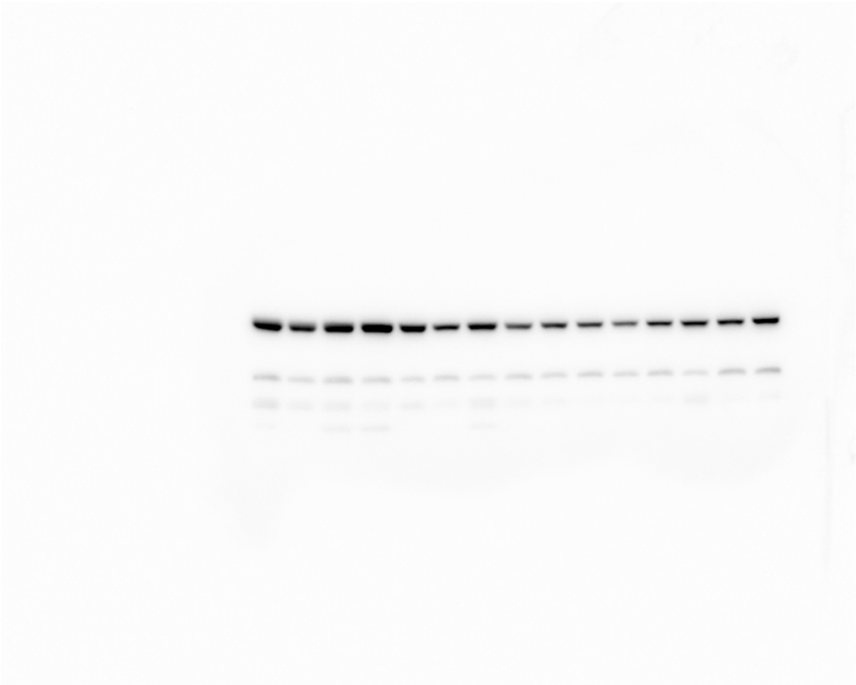

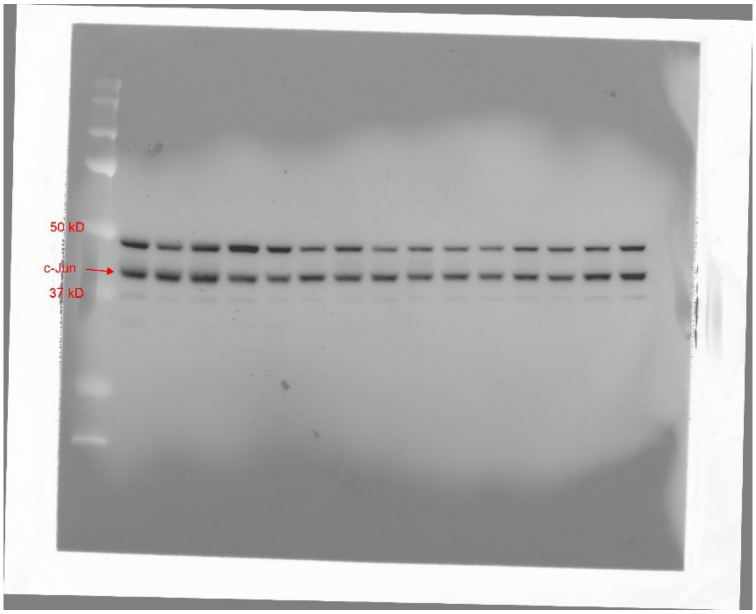
50 kD
c-Jun
37 kD

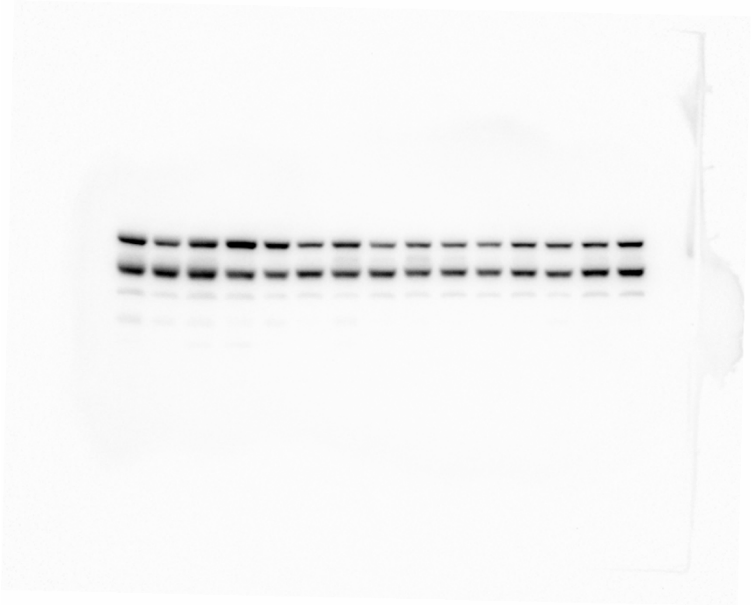

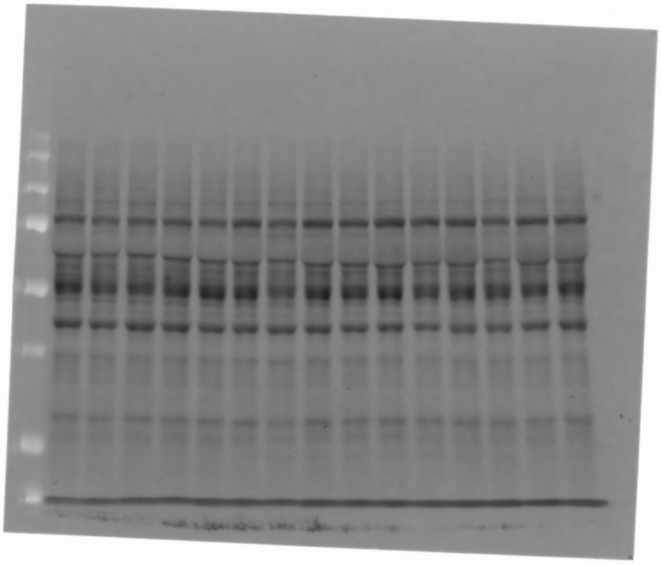

Total lung membrane protein for 28-week-old rats (prevention phase) which was probed for: p-SAPK/JNK, and p-p38.

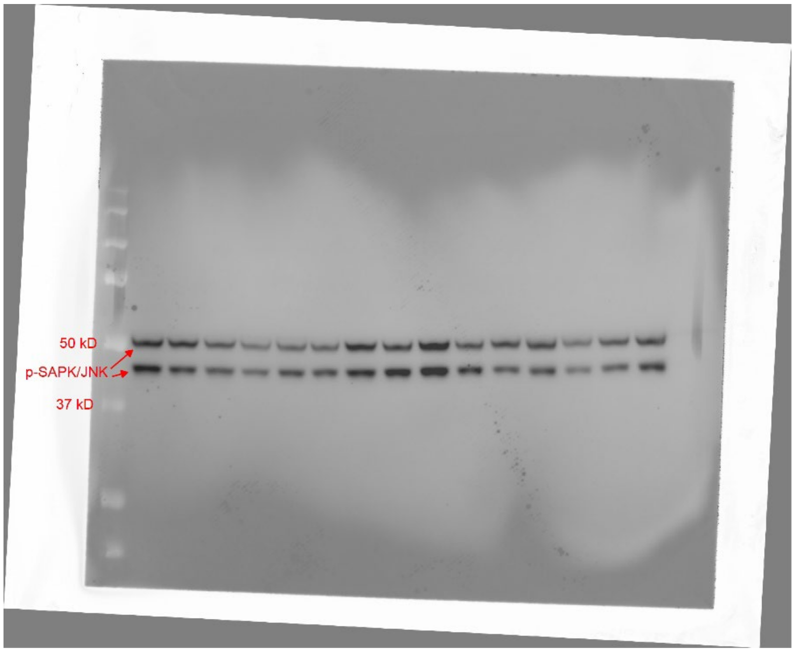
50 kD
p-SAPK/JNK
37 kD

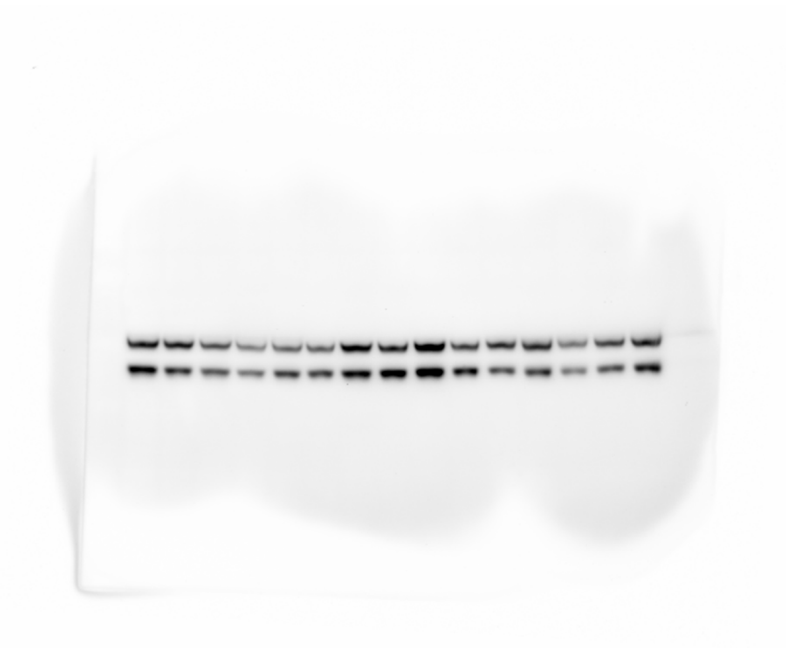

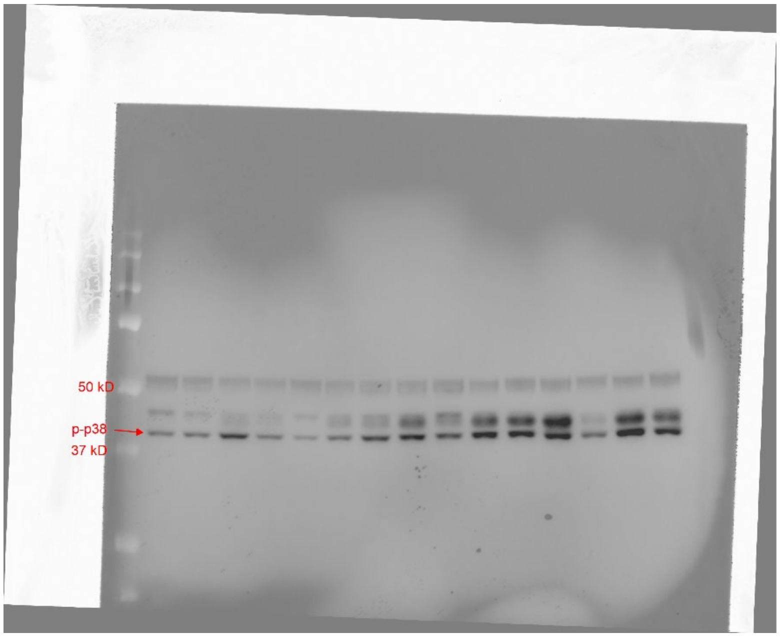
50 kD
p-p38
37 kD

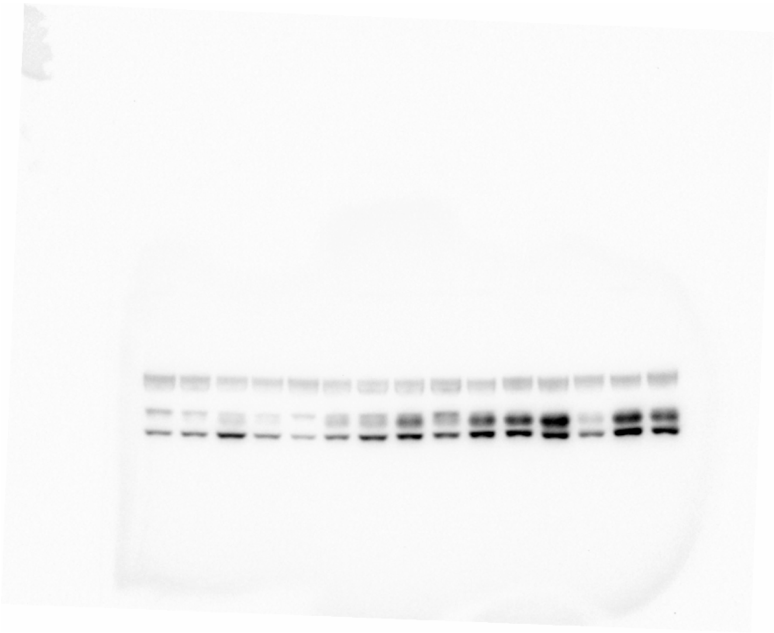

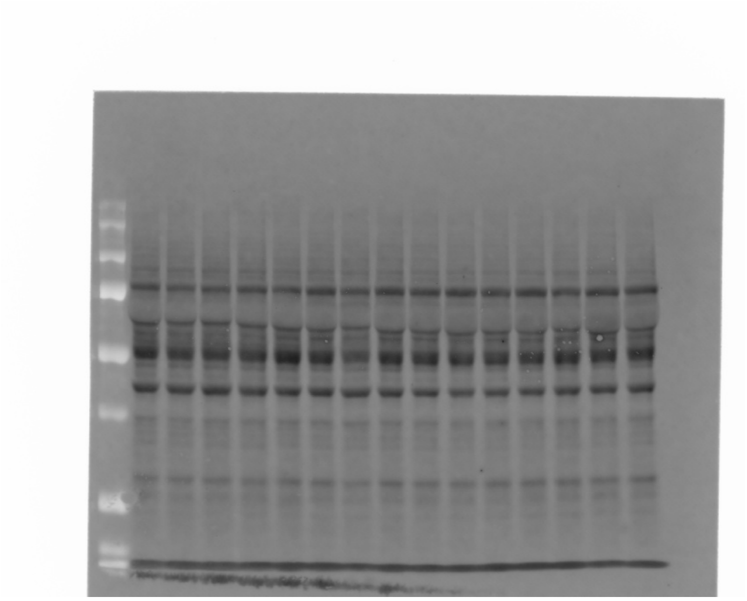

Total lung membrane protein for 28-week-old rats (prevention phase) which was probed for: p-c-Jun, p22phox, and XO.

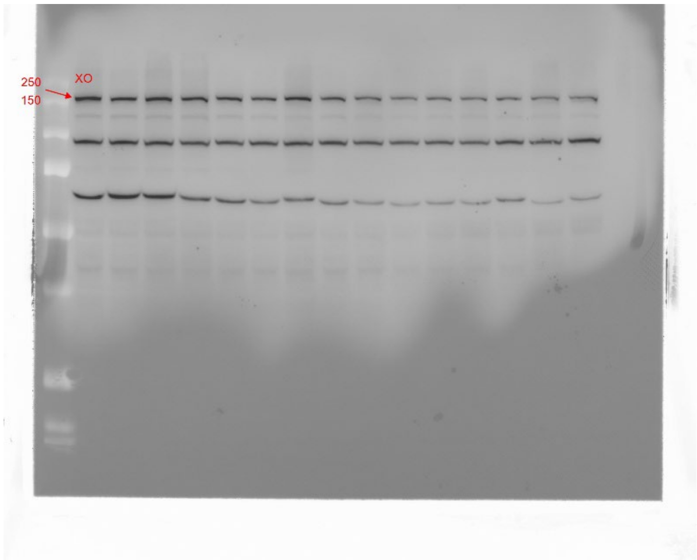
250
150
XO

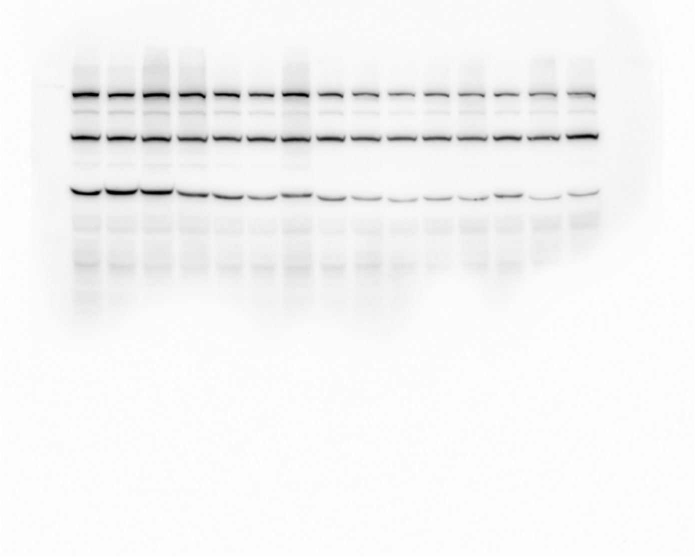

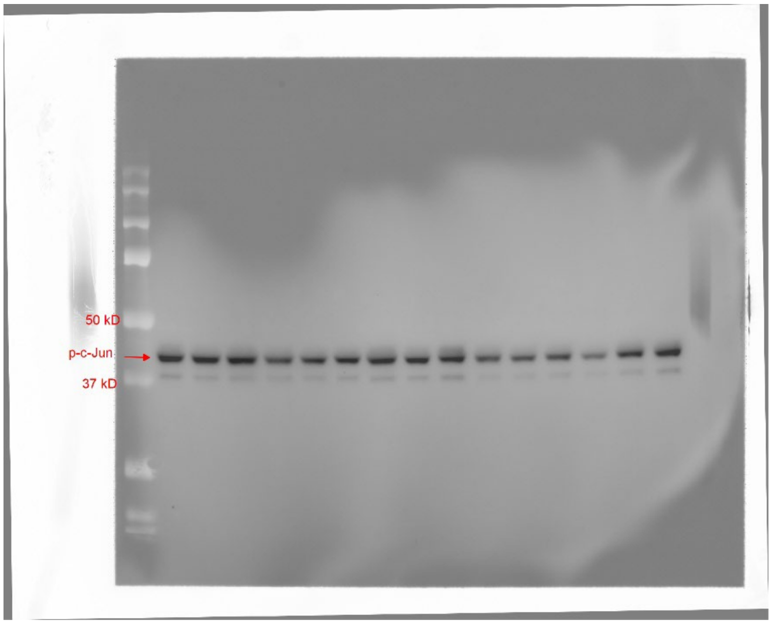

50 kD
p-c-Jun
37 kD

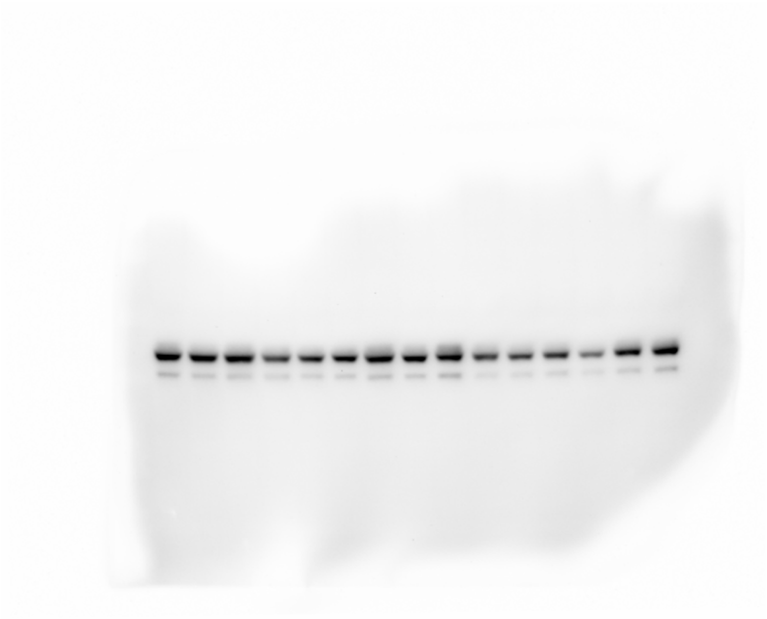

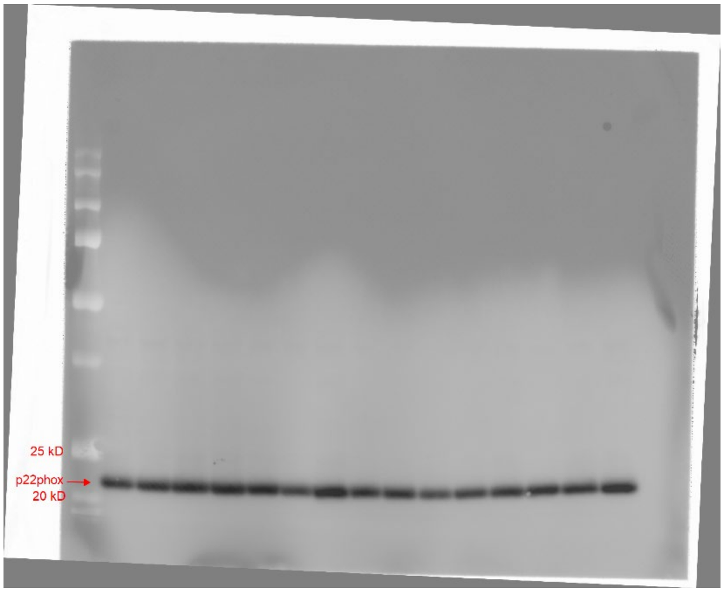
25 kD
p22phox
20 kD

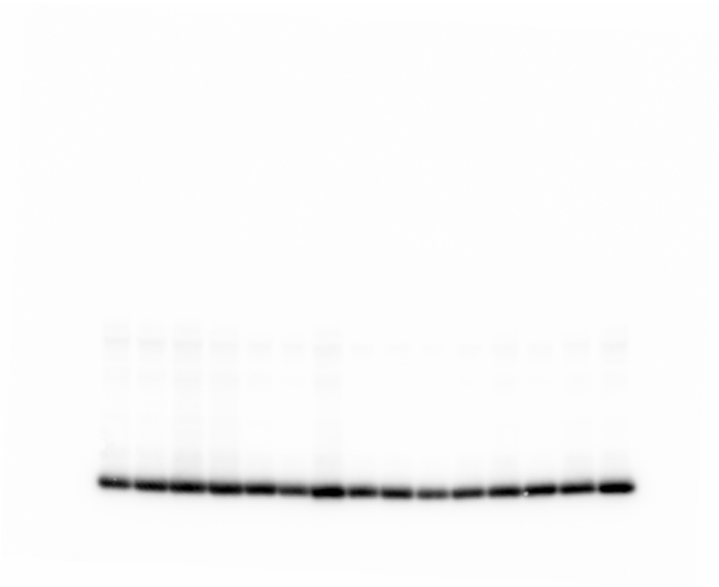

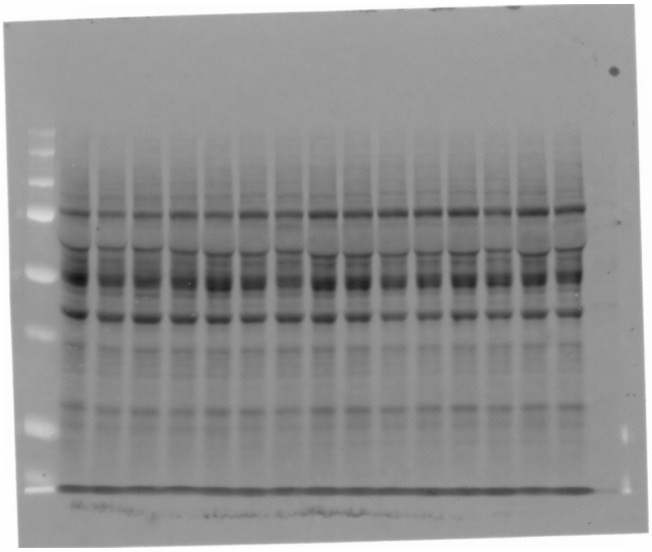

Total lung membrane protein for 28-week-old rats (prevention phase) which was probed for: SAPK/JNK and GPx1.

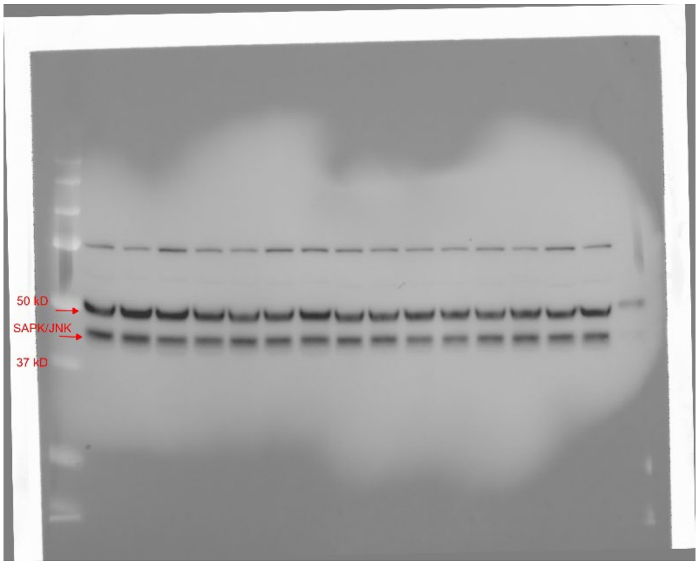
50 kD
SAPK/JNK
37 kD

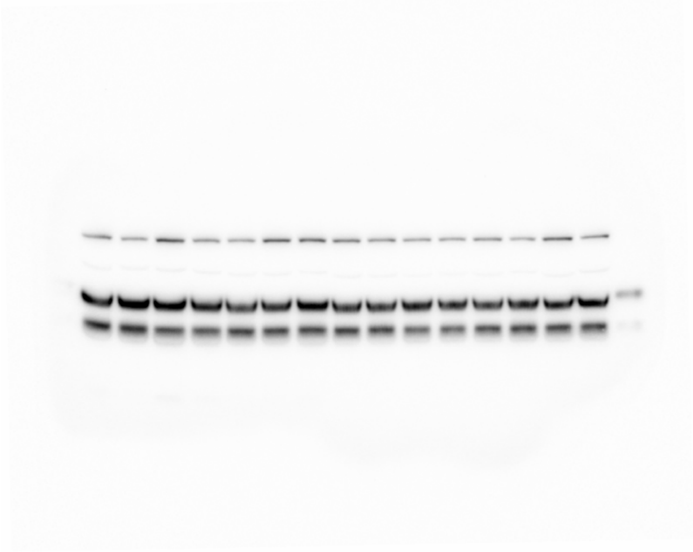

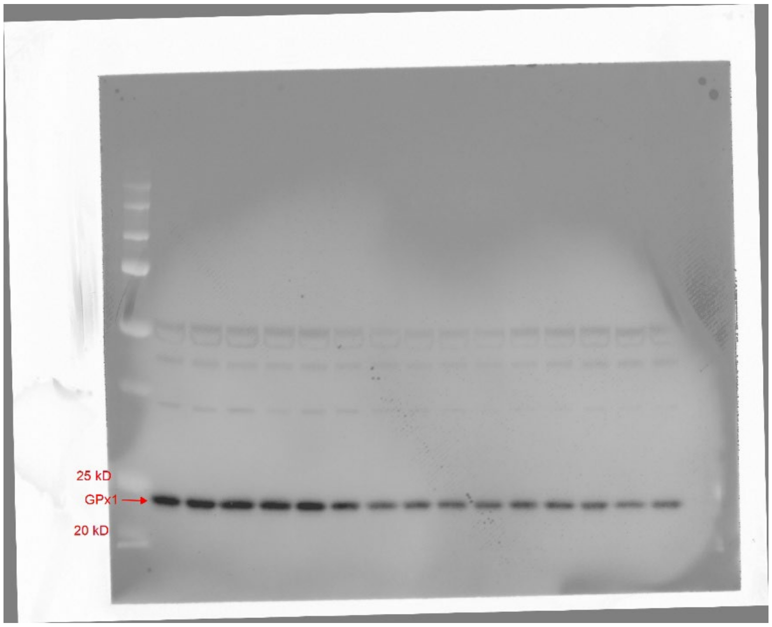
25 kD
GPx1
20 kD

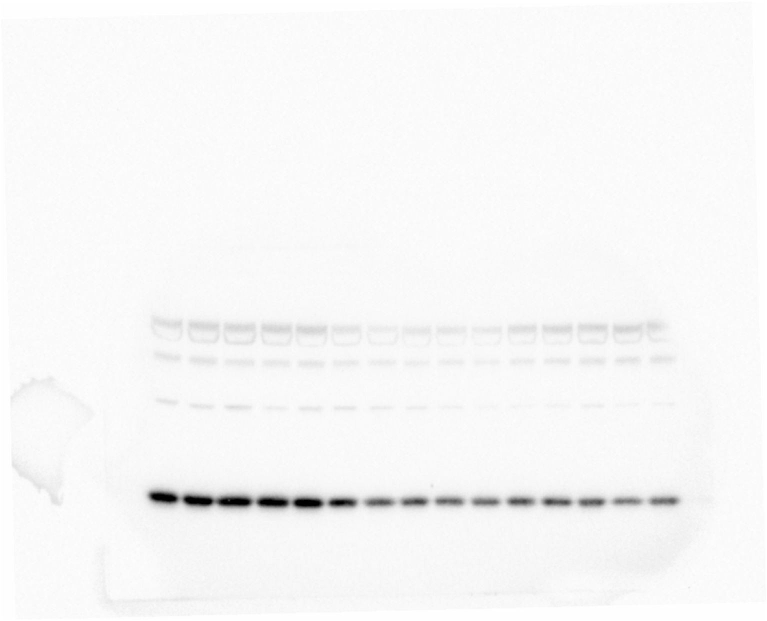

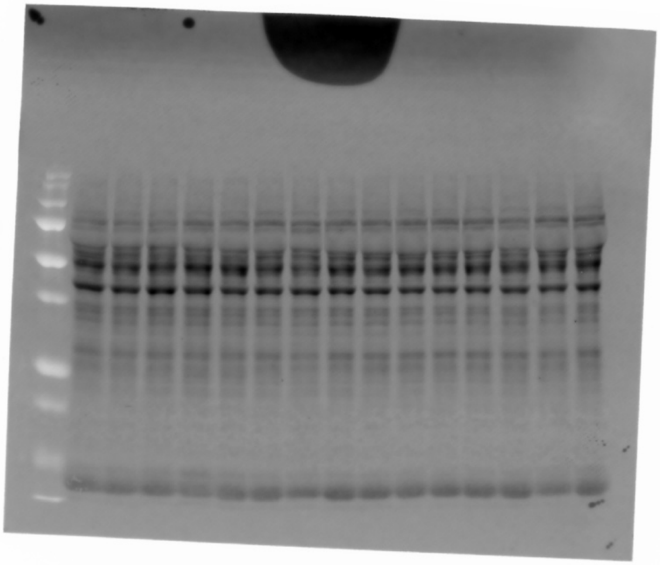

Total lung membrane protein for 28-week-old rats (prevention phase) which was probed for: cleaved TGF-β1, pro-TGF-β1, SOD2, SOD1 and p-NF-κB.

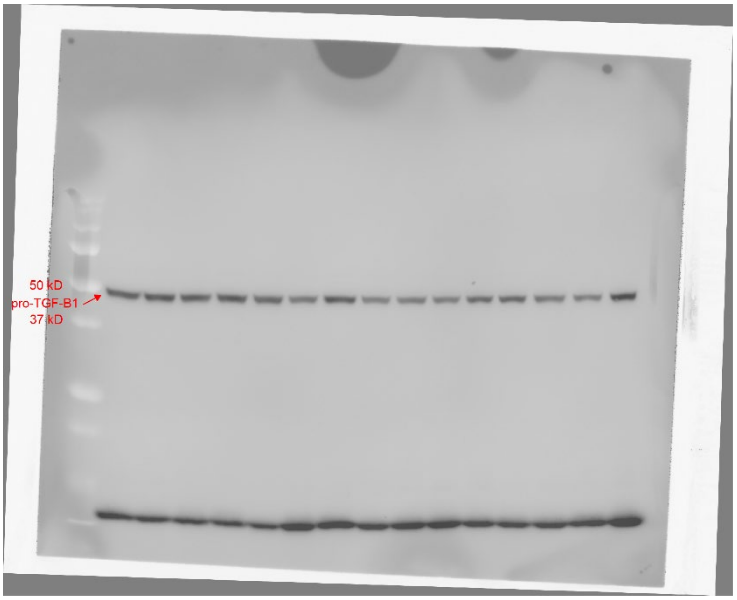
50 kD
pro-TGF-B1
37 kD

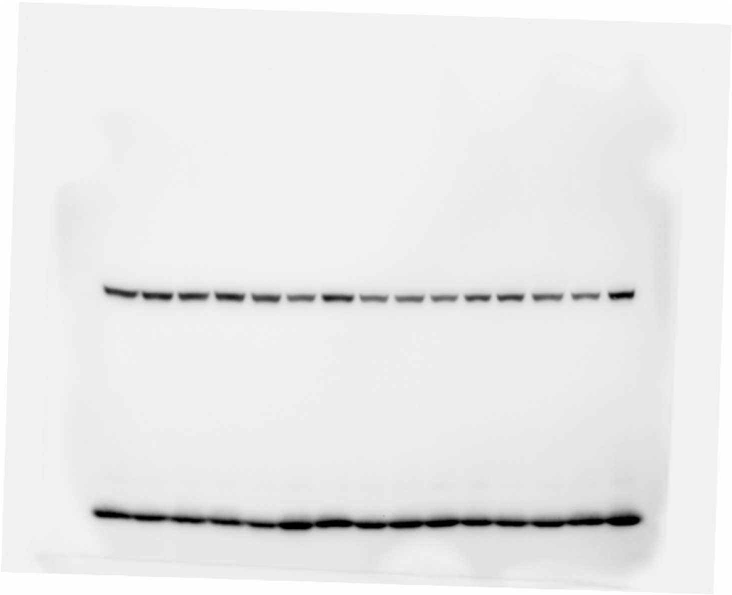

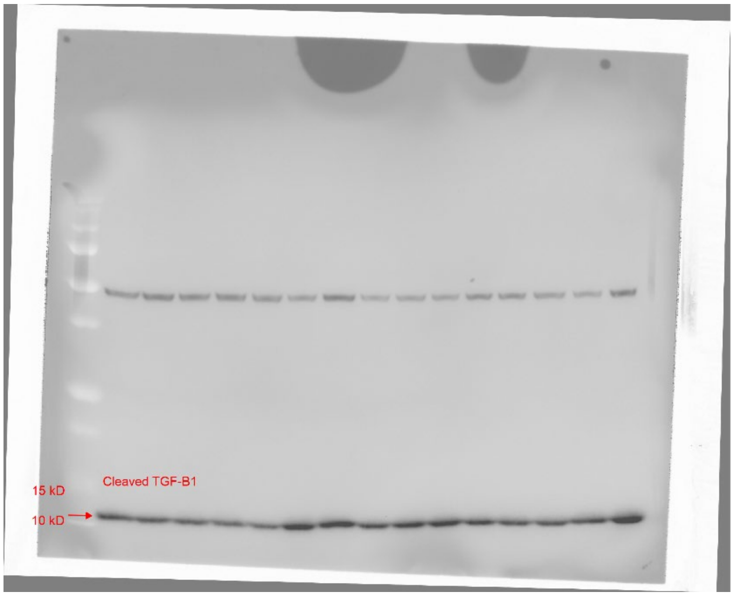
Cleaved TGF-B1
15 kD
10 kD

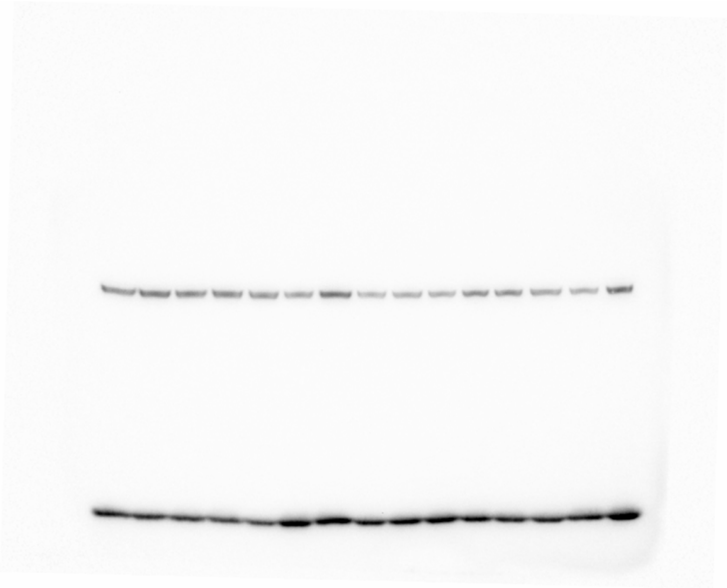

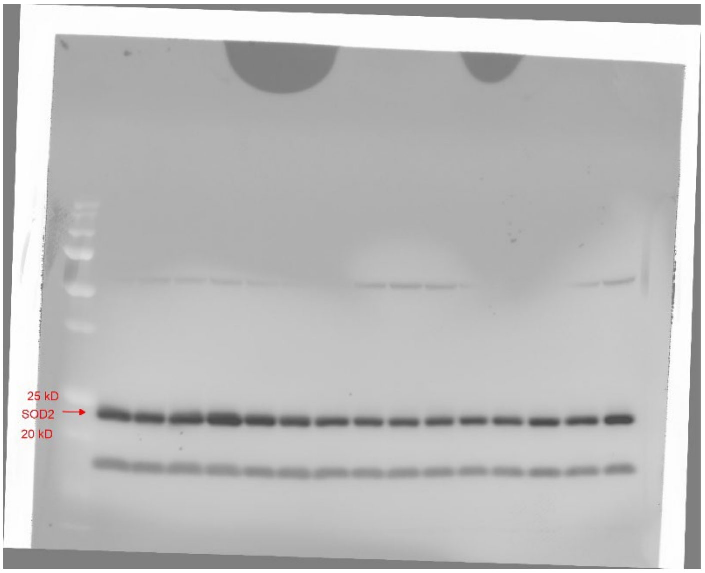
25 kD
SOD2
20 kD

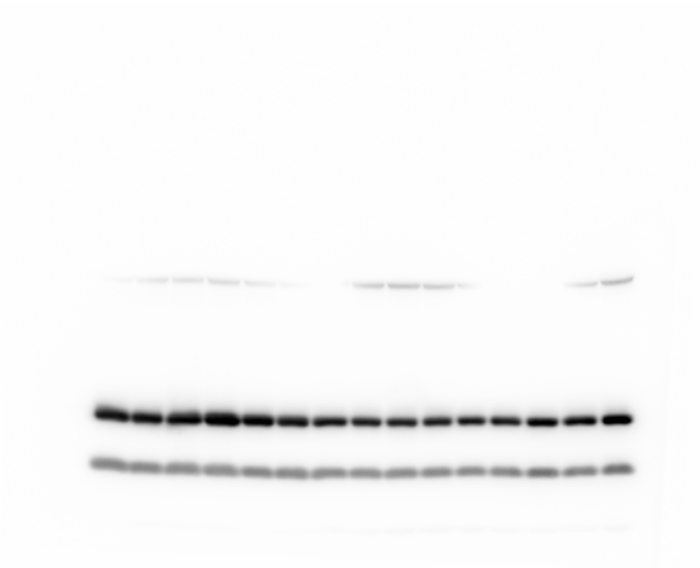

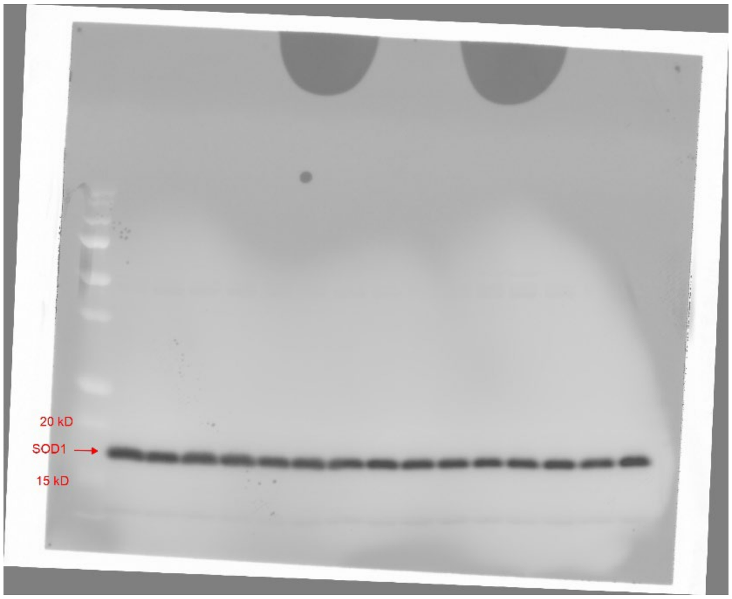

20 kD
SOD1
15 kD

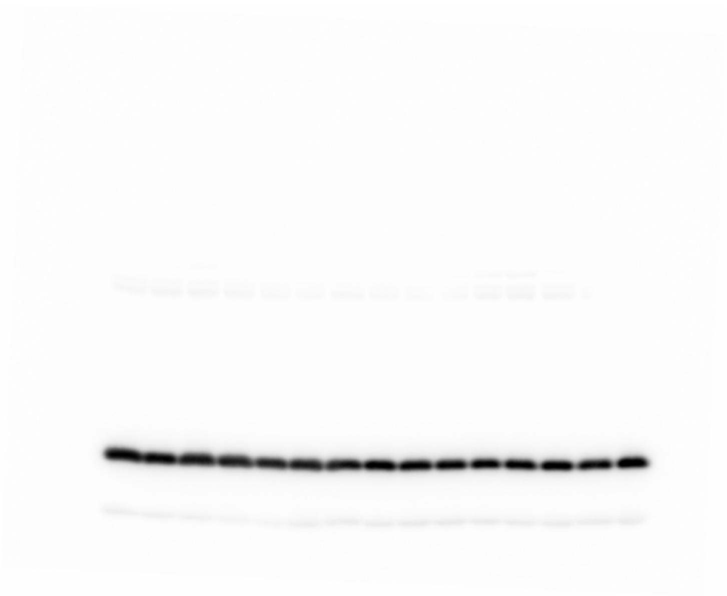

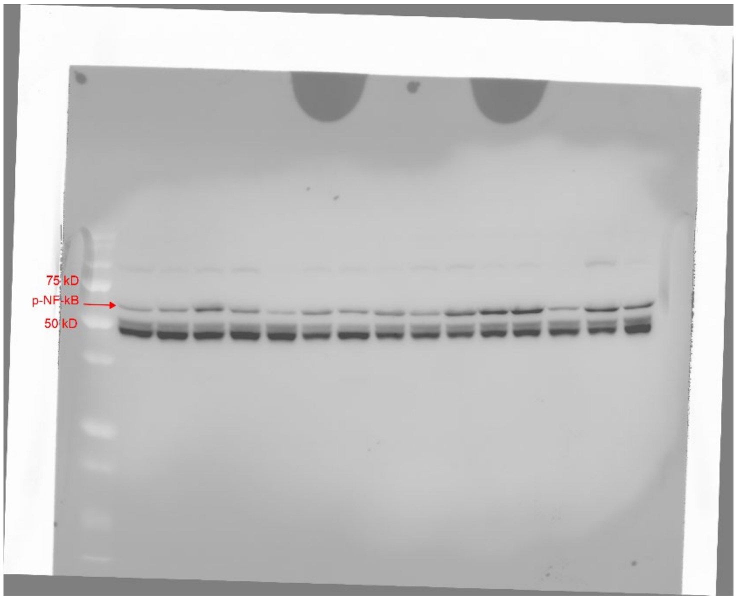
75 kD
p-NF-kB
50 kD

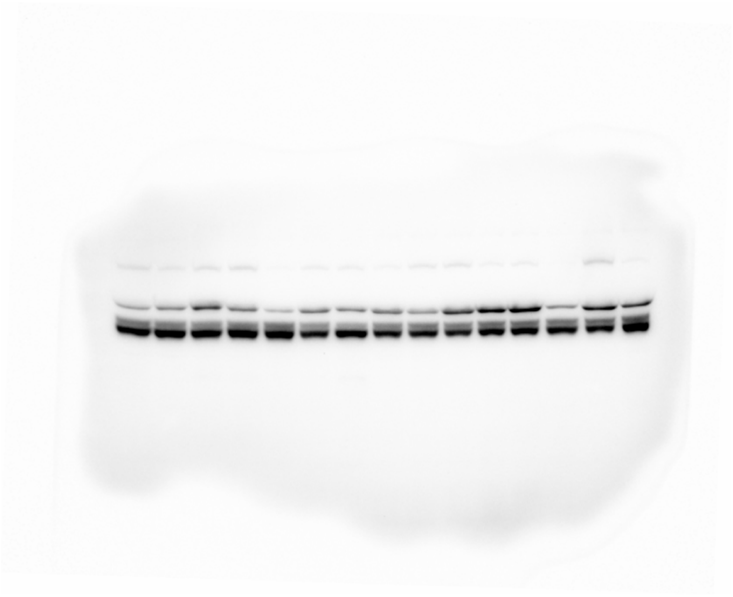

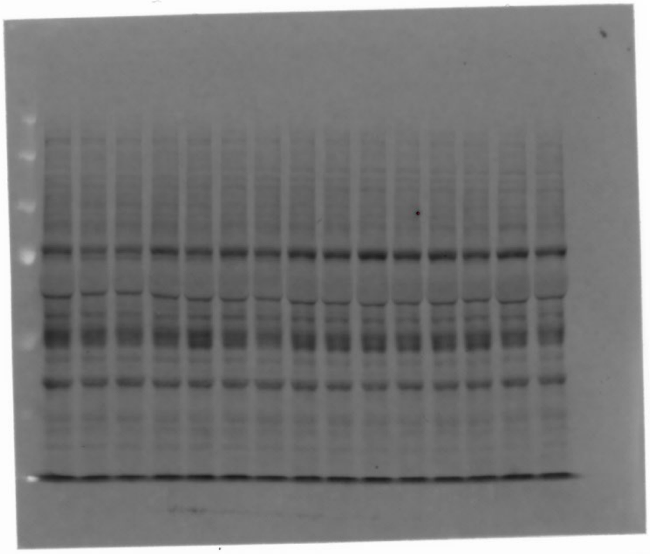

Total lung membrane protein for 28-week-old rats (prevention phase) which was probed for: p-ERK1/2, ERK1/2, NF-κB, F4/80, and E-Cadherin.

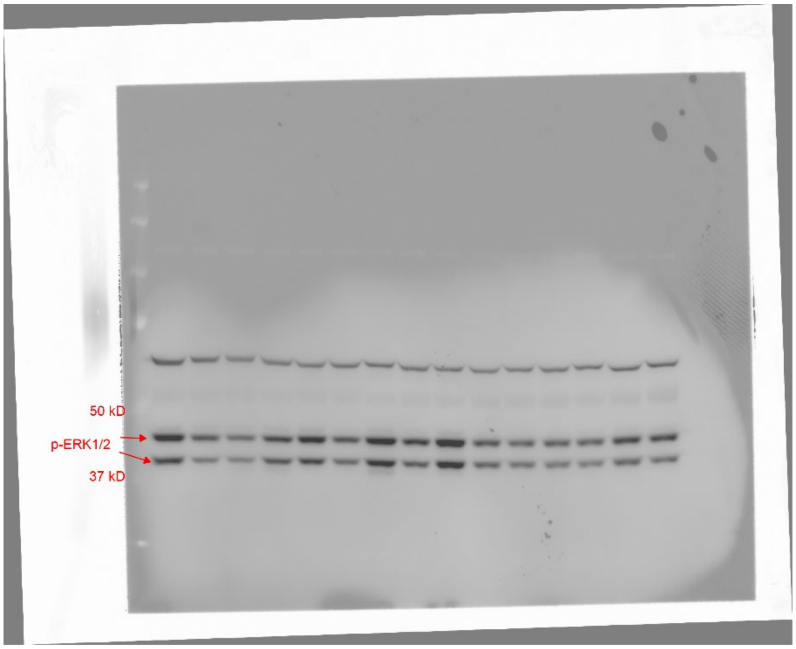
50 kD
p-ERK1/2
37 kD

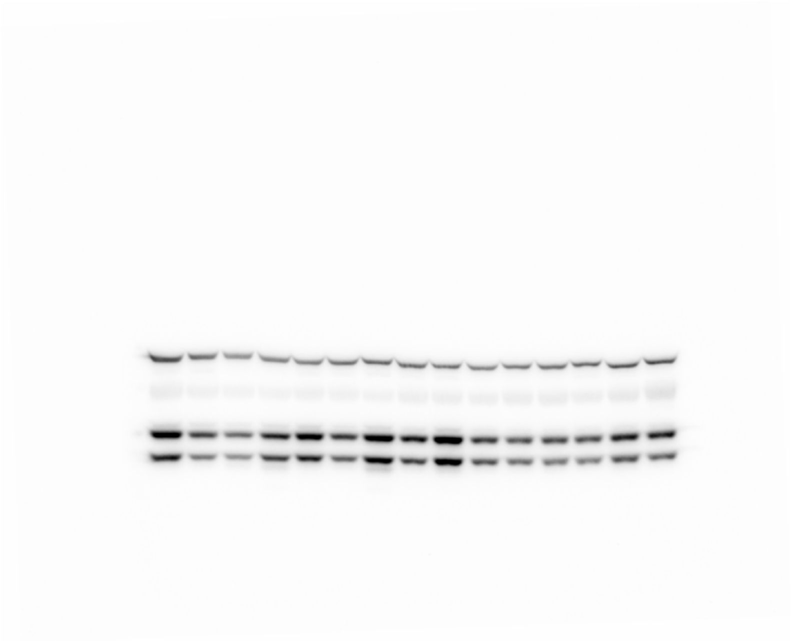

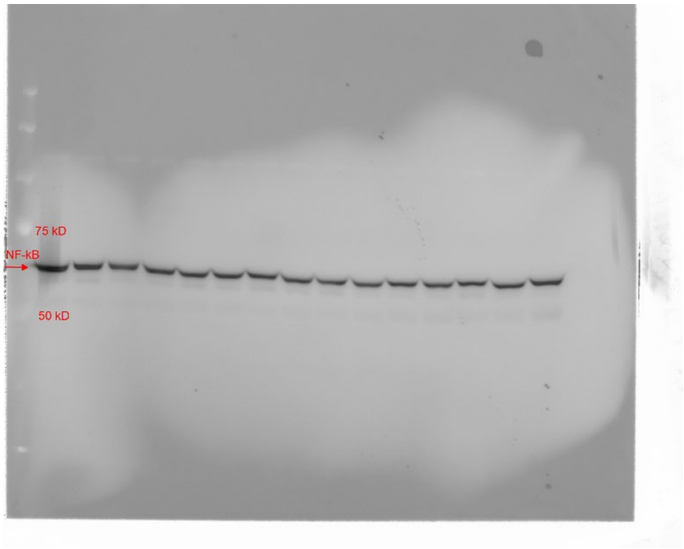
75 kD
NF-kB
50 kD

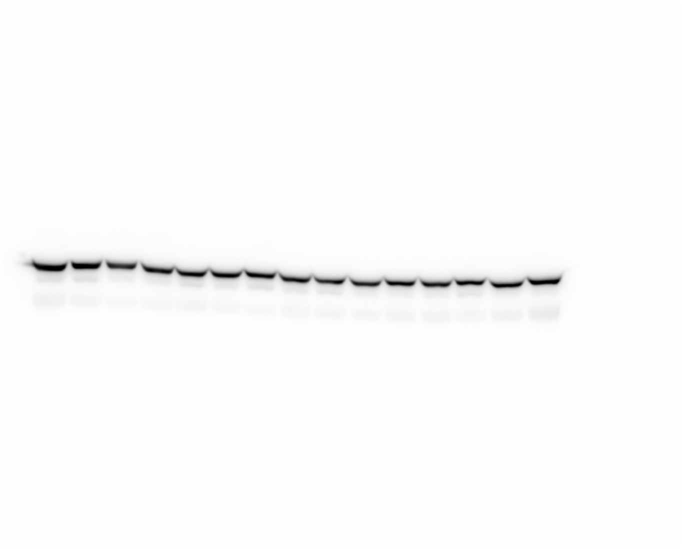

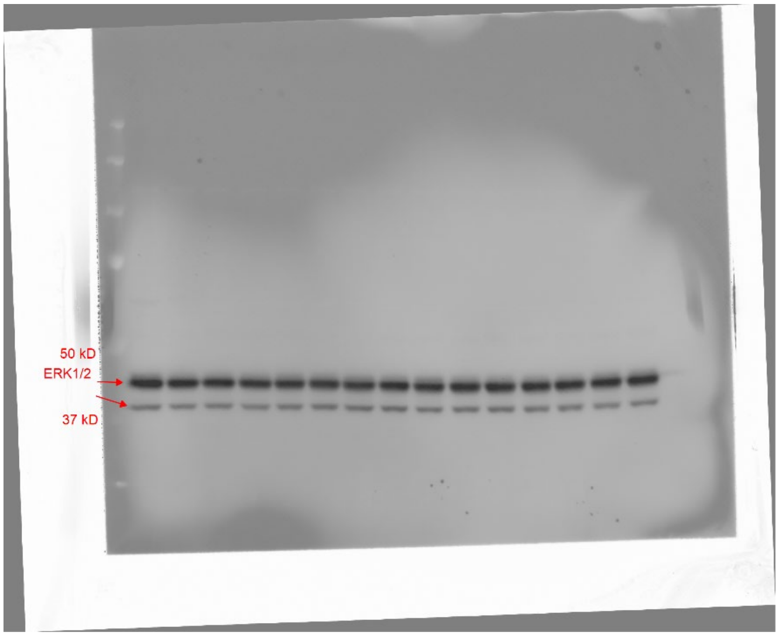
50 kD
ERK1/2
37 kD

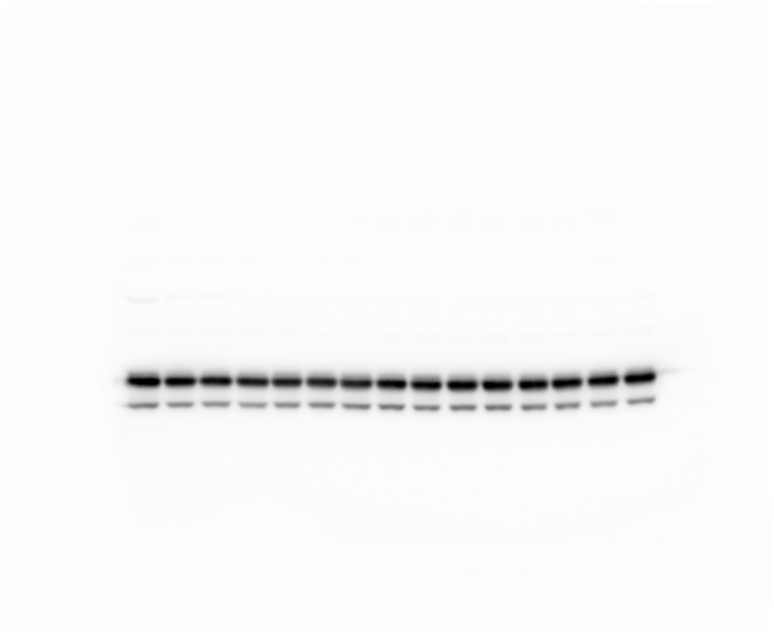

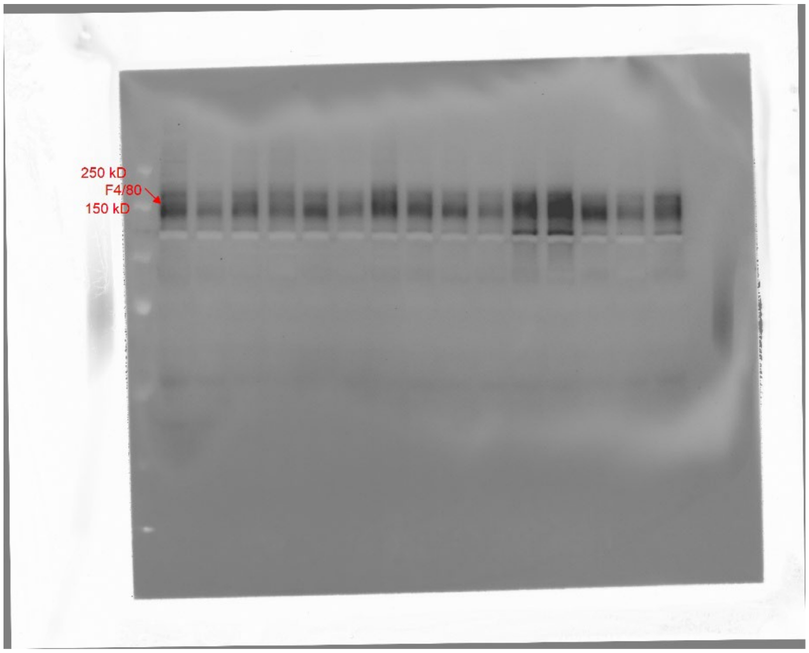
250 kD
F4/80
150 kD

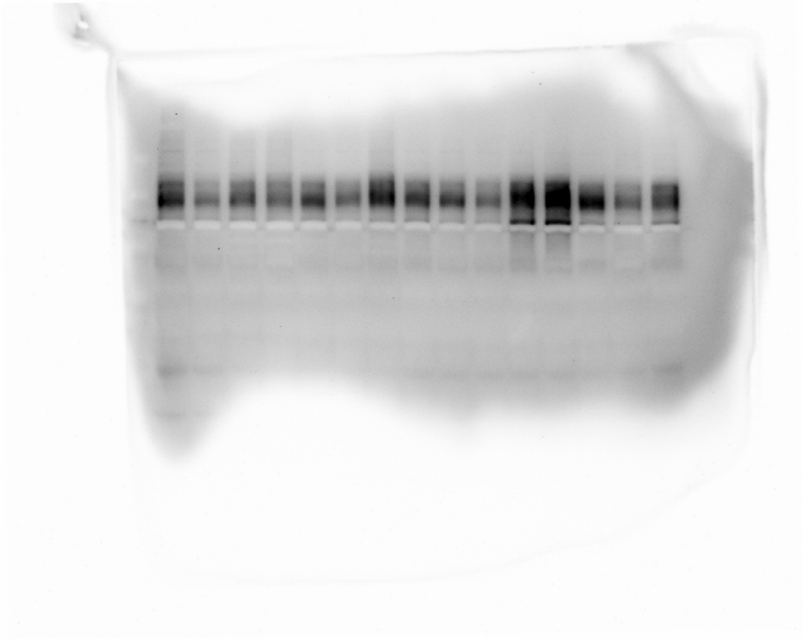

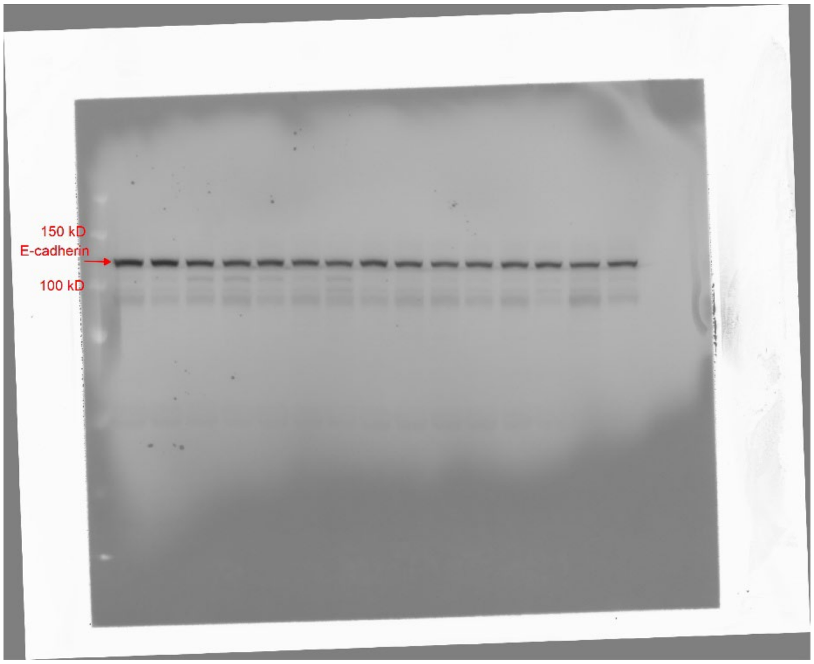
150 kD
E-cadherin
100 kD

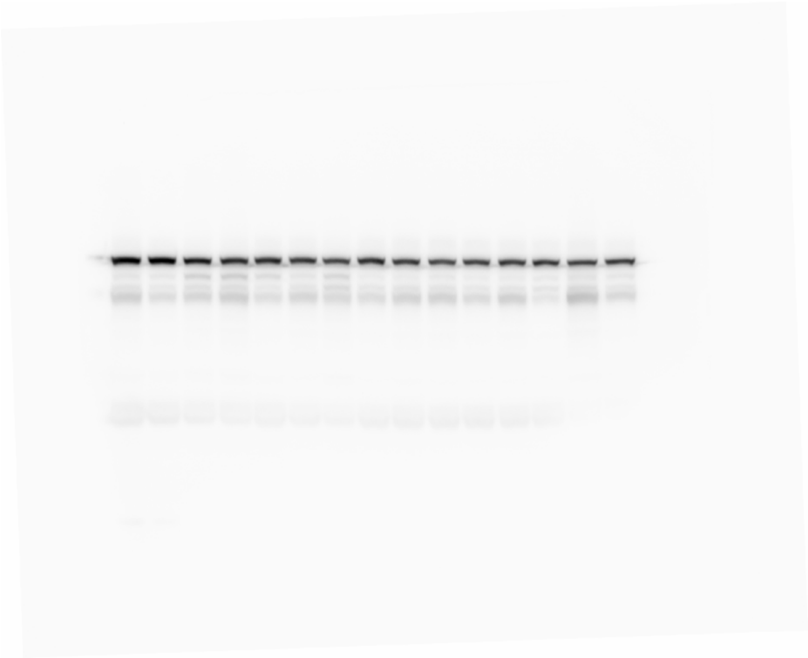

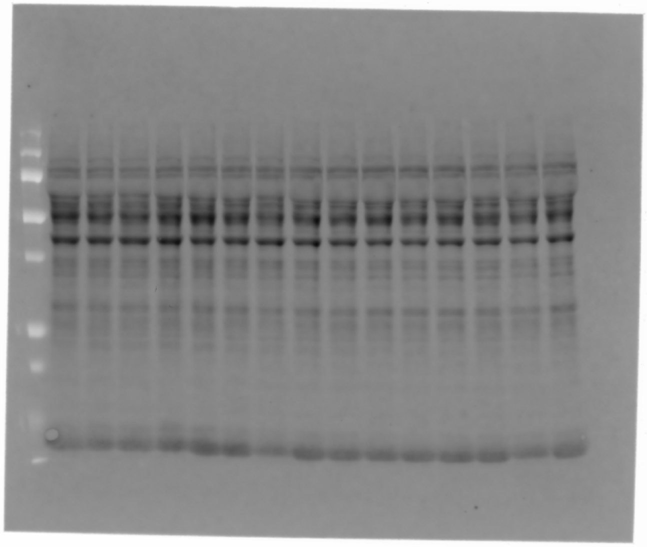

Total lung membrane protein for 28-week-old rats (prevention phase) which was probed for: Vimentin, p38, 3-NT, and α-SMA.

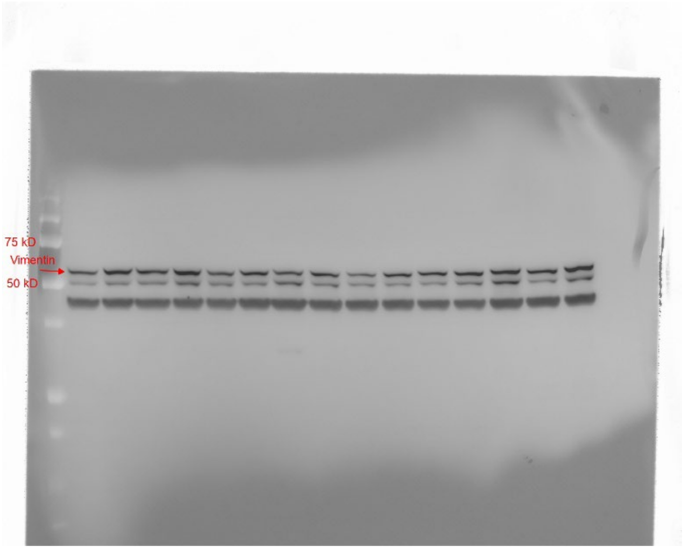
75 kD
Vimentin
50 kD

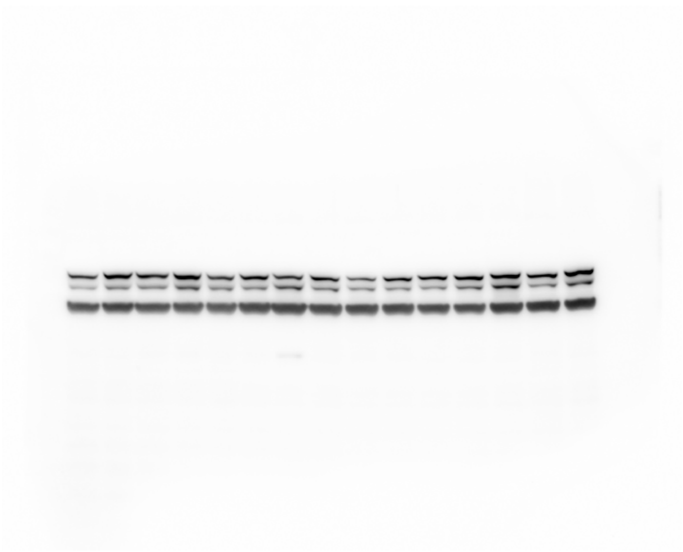

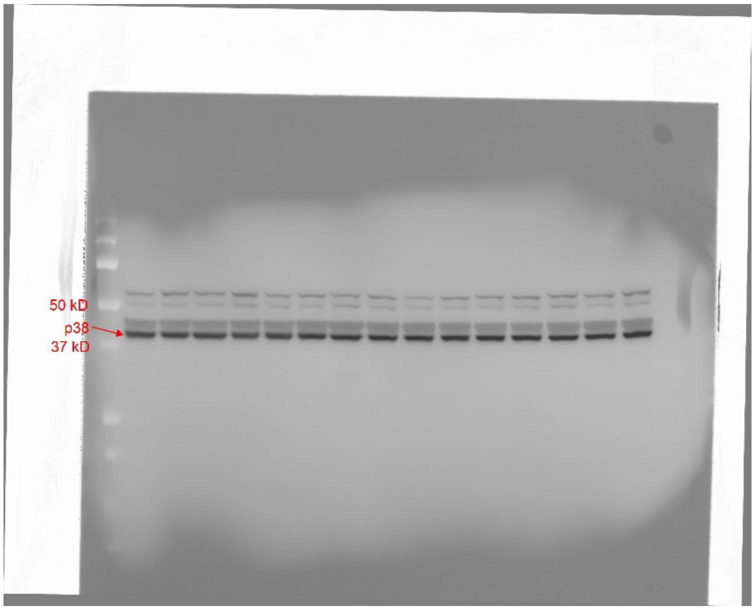
50 kD
p38
37 kD

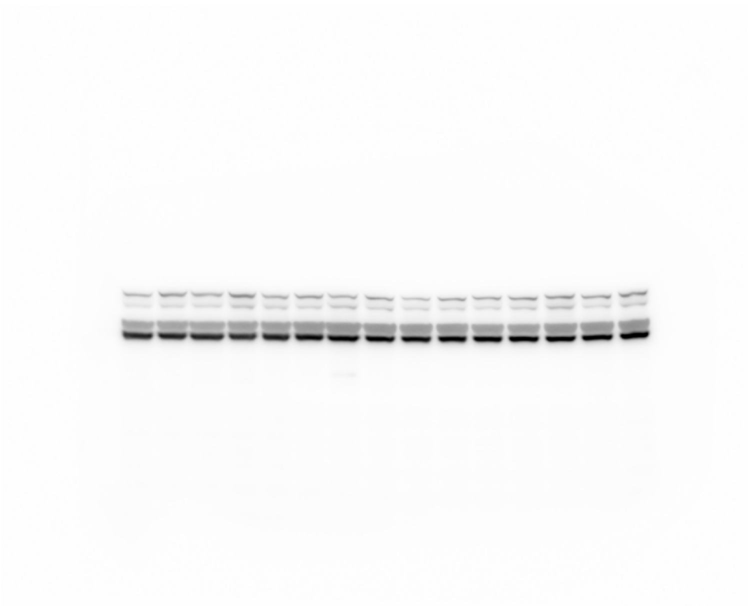

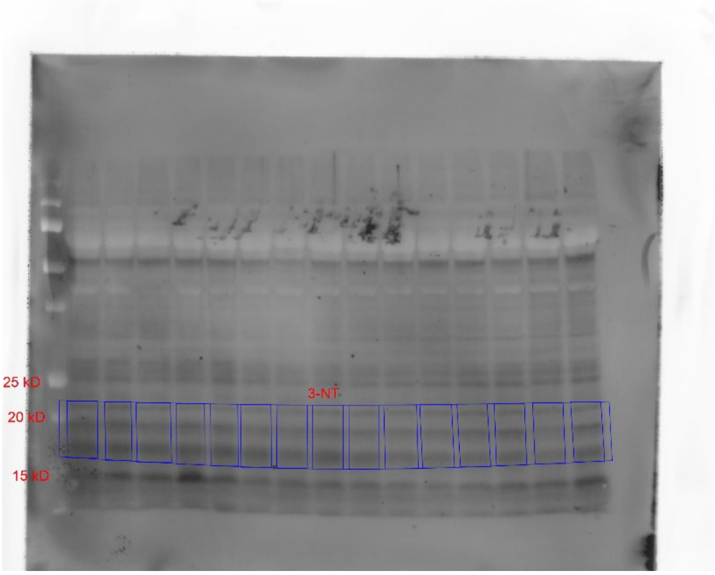
25 kD
3-NT
20 kD
15 kD

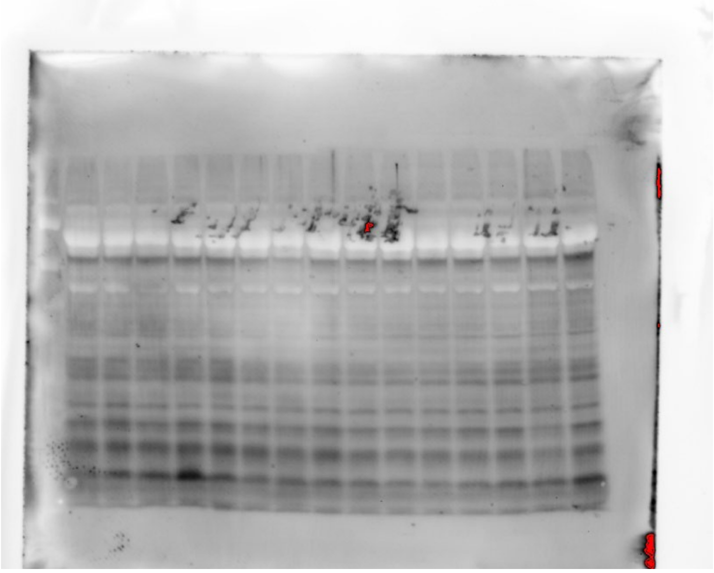

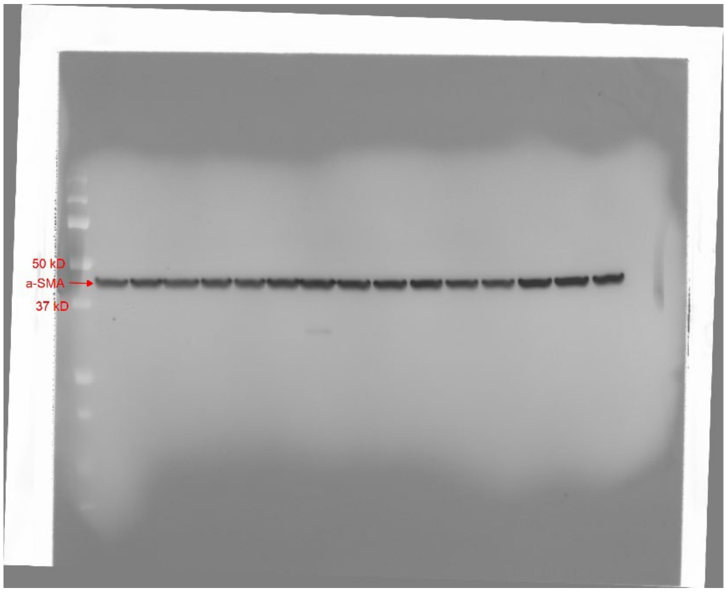
50 kD
a-SMA
37 kD

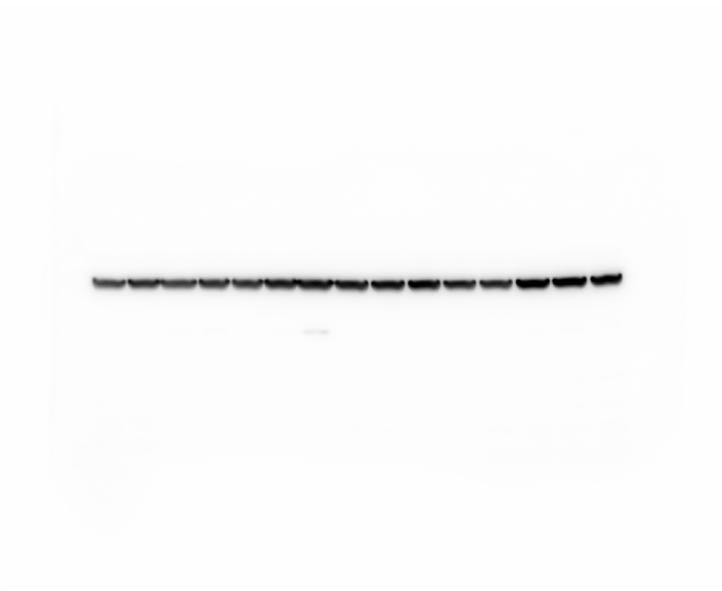

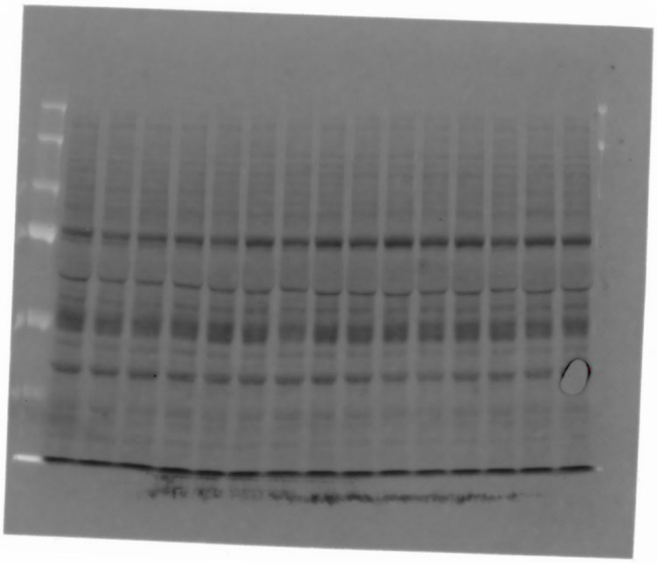

Total lung membrane protein for 28-week-old rats (prevention phase) which was probed for: NRF2, MMP-9, eNOS, and CAT.

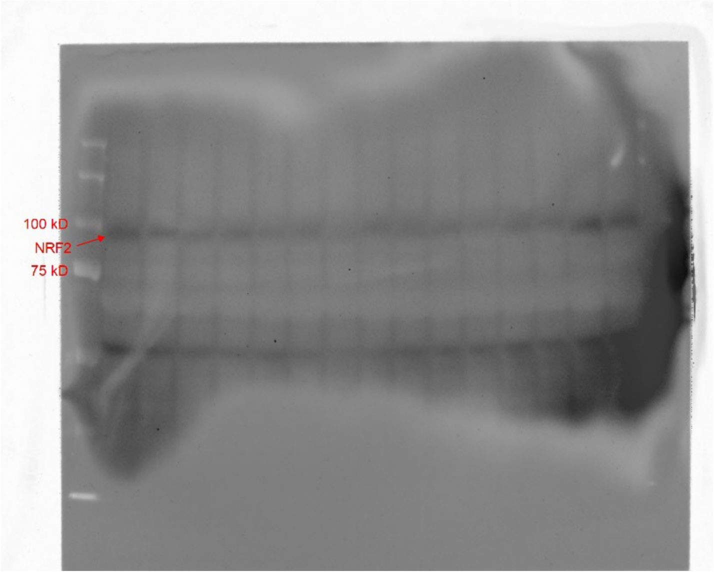
100 kD
NRF2
75 kD

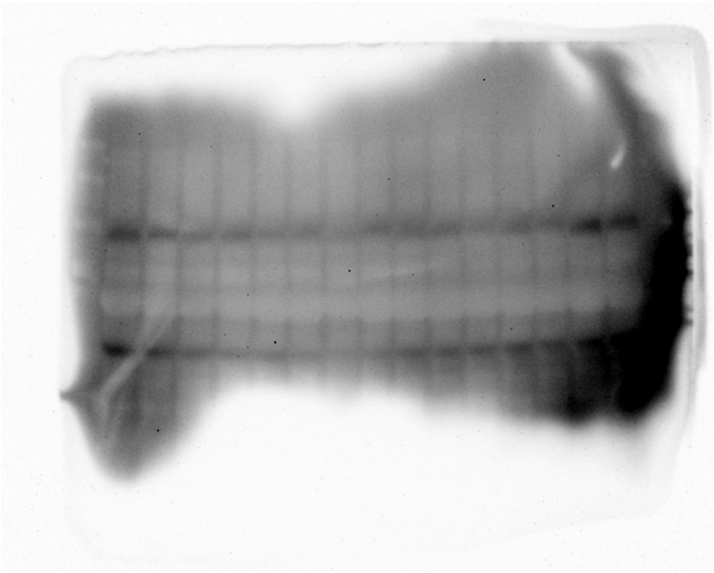

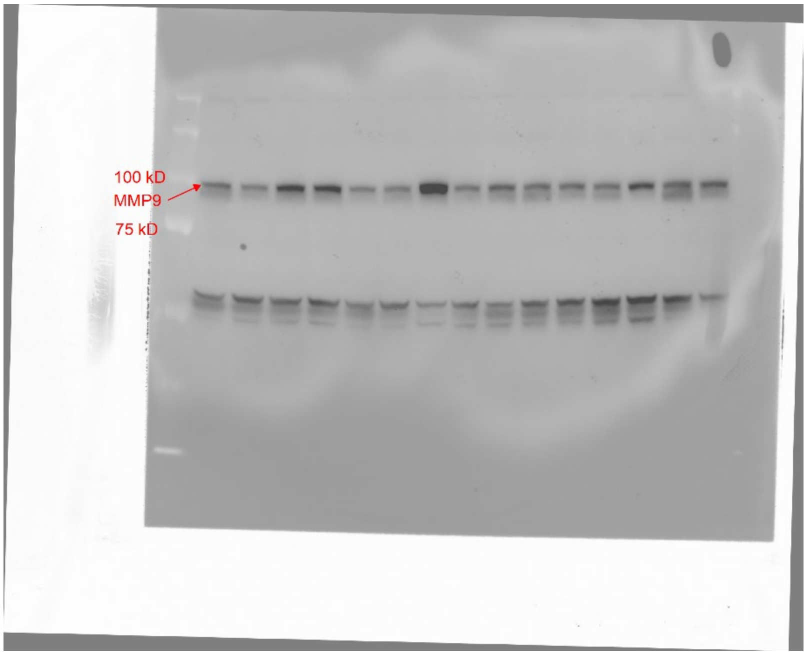
100 kD
MMP9
75 kD

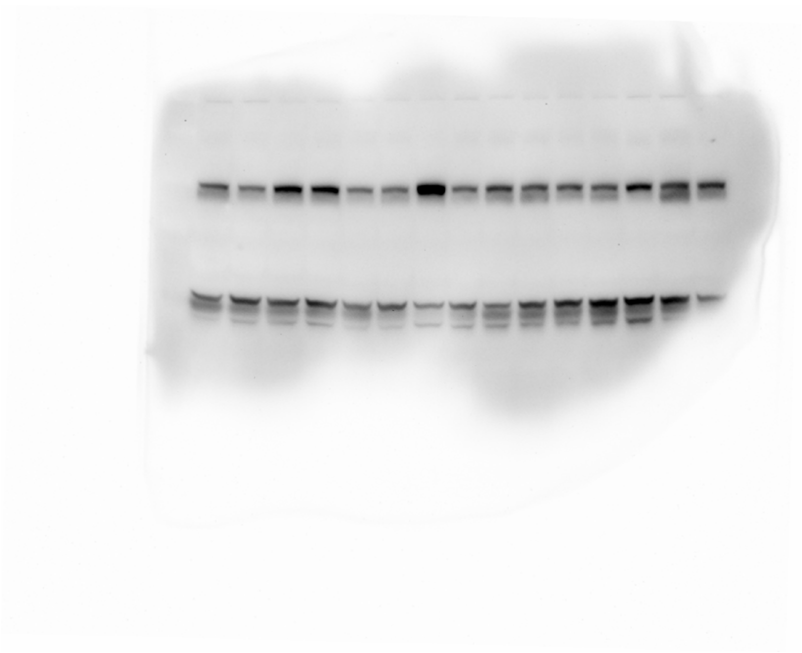

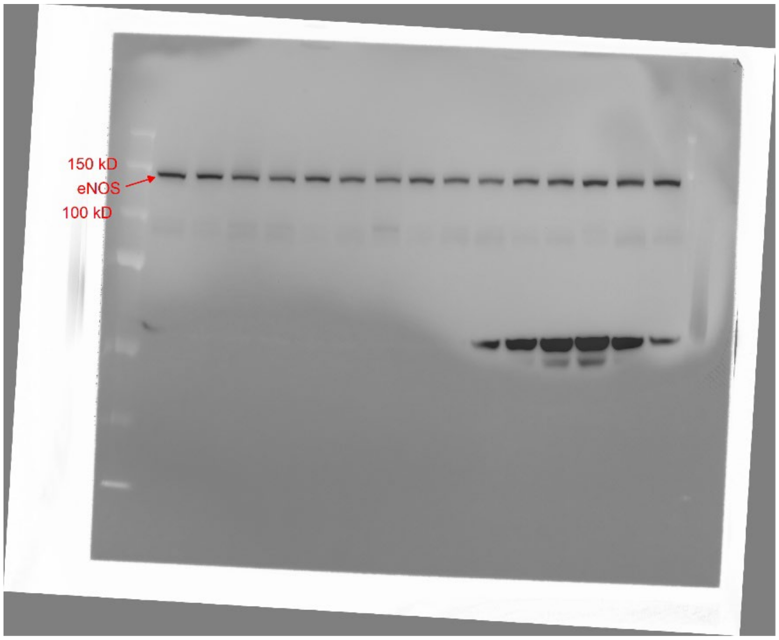
150 kD
eNOS
100 kD

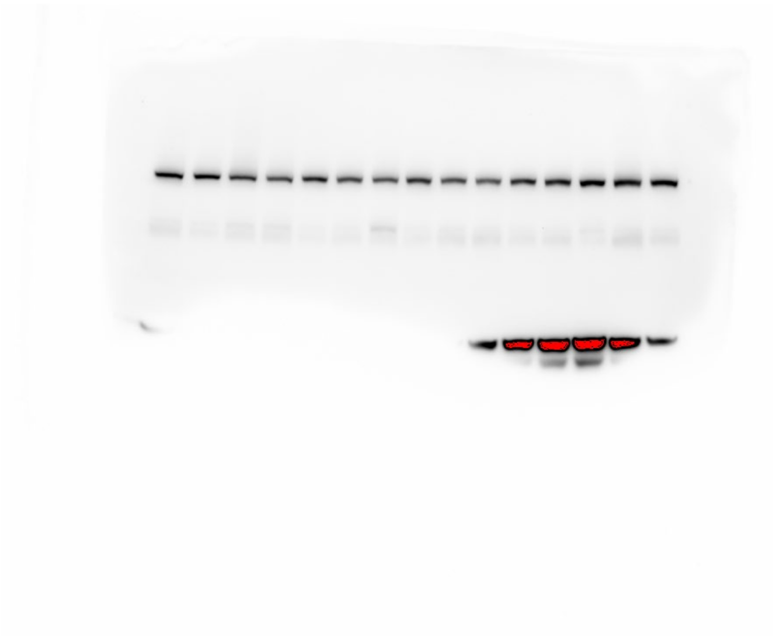

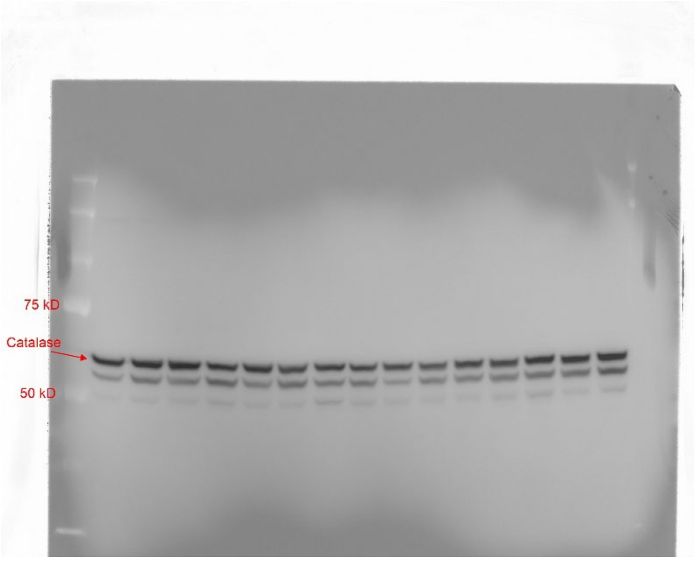
75 kD
Catalase
50 kD

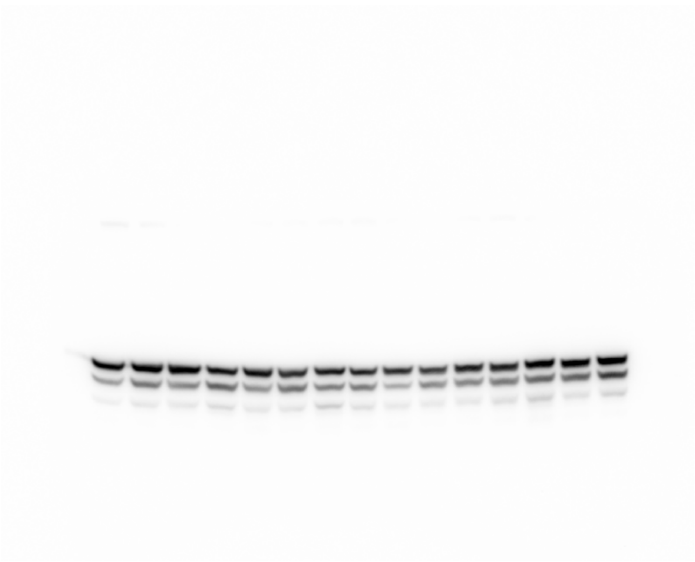

Supplement: Supplementary file 1 [file arm-93-00049-s001.zip › Supplementary materials_raw blots.pdf]
