# Supplementary material for: A Plant-Based Diet Alleviates Molecular Pulmonary Abnormalities in Hypertension
Source: Adv Respir Med. 2025 Nov 4;93(6):49. doi: 10.3390/arm93060049 (PMC12641640; doi:10.3390/arm93060049)
Supplement: Supplementary file 1 [file arm-93-00049-s001.zip › Supplementary Table S1.pdf]

Supplementary Table S1. Composition of diets

	Control diet (D23061303)	Plant-based diet (D23061304)
**Ingredient**	**Grams**	**Grams**
Casein	200	0
Soy protein	0	153
Methionine	0	2.5
L-Cystine	3	0
Corn starch	252.5	137.6
Maltodextrin 10	150	150
Dextrose	150	150
Sucrose	102.41	100
Cellulose	75	9.2
Inulin	25	2.9
Soybean oil	70	32.3
Mineral Mix S10026 (RD-96)	0	10
Mineral Mix S10026A (RD-96 w/o NaCl)	5	0
DiCalcium Phosphate	15.4	13
Calcium Carbonate	11	5.5
Potassium Citrate, 1 H2O	29	16.5
Sodium Chloride	4.07	0
Copper Carbonate	0.0071	0
Ferric Citrate	0.0740	0
Zinc Carbonate	0.022	0
Vitamin Mix V10001 (76A)	0	10
Vitamin Mix V15937 (Special Vit. Mix)	10	0
Choline Bitartrate	2	2
Plant mix	0	309
	**Gram %**	**Gram %**
Protein	16	16
Carbohydrate	59.3	59.4
Fat	6.6	6.6
Insoluble fiber	6.8	6.8
Soluble fiber	2.3	2.3
Plant mix	0	28
